# Measurement of fatigue in sickle cell disease: a systematic review of fatigue measures

**DOI:** 10.1186/s13023-025-03961-4

**Published:** 2025-09-03

**Authors:** Alice Gourdin, Damien Oudin Doglioni, Michalina Dannoune, Mélanie Astié, Fanny Hamelin, Sébastien Monnier, Caroline Makowski, Marie-Claire Gay

**Affiliations:** 1 Laboratoire Interuniversitaire de Psychologie/Personnalité, Cognition, Changement Social (LIP/PC2S), Univ. Grenoble Alpes, Univ. Savoie Mont Blanc, Grenoble, France; 2 Centre de Compétence Filière Santé Maladies Rares MCGRE « Maladies Constitutionnelles du Globule Rouge et de L’Erythropoïèse », Médecine Interne, Maladies Infectieuses et Tropicales, CH de Versailles, Le Chesnay, France; 3 Centre de Compétence Filière Santé Maladies Rares MCGRE « Maladies Constitutionnelles du Globule Rouge et de L’Erythropoïèse », Médecine Interne, Centre Hospitalo-Universitaire Grenoble Alpes, Grenoble, France; 4 Structure Régionale Neuro SEP SYNAPSE (ex Réseau SEP IDF Ouest), Hôpital du Vésinet, Vésinet, France; 5 Évaluation Clinique des Psychothérapies et de La Psychopathologie (EVACLIPSYD), Université Paris Nanterre, Nanterre, France

**Keywords:** Fatigue, Sickle cell disease, Measurement tool, Fatigue dimensions

## Abstract

**Background:**

Sickle cell disease (SCD) is a chronic inherited blood disorder caused by abnormal haemoglobin production, affecting over seven million people worldwide. Although pain—particularly acute bone pain—is the hallmark symptom of this disease, fatigue is also a commonly observed manifestation. Fatigue is a debilitating symptom in Sickle Cell Disease (SCD) that significantly impacts quality of life. Accurate assessment of fatigue is crucial for effective disease management. However, a comprehensive analysis of fatigue assessment tools in SCD research is lacking.

**Objective:**

This systematic literature review aims to identify and evaluate self-reported psychometric measures of fatigue used in SCD research with children, adolescents, young adults and adults.

**Methods:**

A systematic search was conducted across six databases from 2010 to March 2024. The main inclusion criteria included peer-reviewed journal articles, patients with all SCD genotypes, studies evaluating fatigue using a self-reported psychometric measure, and studies published in English or French. The PRISMA guidelines were followed for study selection and data extraction.

**Results:**

Twenty-eight studies met the inclusion criteria, reporting on 16 psychometric measures of fatigue. The most frequently used tool was the PROMIS system. Nine dimensions of fatigue were identified, including general, physical, mental, cognitive, emotional fatigue, and its impact on motivation, activity, vigour, and sleep/rest. However, the definitions of these dimensions were often unclear. Reported fatigue scores are not directly comparable due to methodological issues and variability in the assessment used. These methodological issues limit our knowledge on the prevalence of fatigue in SCD.

**Conclusion:**

The lack of a standardised fatigue assessment tool in SCD research hinders direct comparison of fatigue scores across studies. Future research should prioritise the development of a tailored assessment tool for SCD, considering the specific dimensions of fatigue relevant to this population. In the interim, clinicians and researchers can employ a combination of multidimensional and unidimensional tools to gain a more comprehensive understanding of patients' fatigue experiences.

**Supplementary Information:**

The online version contains supplementary material available at 10.1186/s13023-025-03961-4.

## Background

Sickle cell disease (SCD) a prevalent inherited blood disorder affecting over seven million individuals worldwide [1], disproportionately impacts populations with African, Mediterranean, Middle Eastern, and South Asian ancestry [2]. In France, SCD represents the most common inherited genetic disease [3], with a reported incidence of one in 1323 births in 2020 [4]. This chronic illness arises from abnormal haemoglobin production [2]. SCD encompasses a spectrum of genotypes, with severity varying considerably based on the presenting symptomatology and disease course [5].

The hallmark of the disease is the erythrocyte sickling property linked to pathological and—to some extent—reversible transient haemoglobin polymerisation under specific conditions. This phenomenon occurs mainly in deoxygenated erythrocytes of blood stream at postcapillary areas. The disease manifests as a spectrum of symptoms and complications, depending roughly on phenotype and severity. Tissular lesions develop over time due to recurrent incidents in the bloodstream, including hyperviscosity, ischaemic and reperfusion phenomena, haemolysis, inflammation, and chronic endothelial dysfunction. Acute pain is the key symptom, as bone is inextensible and highly sensitive; however, several mechanisms are involved in silent diffuse tissue lesions, leading to organ damage and dysfunction. Chronic anaemia is common, associated with recurrent acute episodes of severe pain (vaso-occlusive crises), chronic pain, cerebral vasculopathy, stroke or silent stroke, sleep disorders, and heightened susceptibility to fatigue [2, 6]. Additionally, patients are prone to infectious complications, mainly linked to splenic function impairment. The influence of this pathology on fatigue can be further explained by its chronic nature punctuated by acute episodes and complications.

Chronic fatigue is a well-documented burden in chronic disease and SCD, significantly impacting individuals’ quality of life [7]. While fatigue is a common experience, it becomes a clinical symptom when it persistently disrupts daily activities and functioning [8]. Several SCD complications, such as chronic haemolytic anaemia, inflammation, and pain, are known contributors to both acute and chronic fatigue [6]. However, the precise nature, underlying mechanisms, and overall prevalence of fatigue in SCD remain areas of ongoing investigation [6, 9].

Despite being recognised alongside pain as a hallmark symptoms of SCD, fatigue remains understudied in the literature [10]. However, recent research are increasingly acknowledging its prevalence, with studies reporting fatigue as a frequent and concerning issue for patients [9–13]. For instance, studies have shown a high prevalence of fatigue, with reports exceeding 65% [9], and fatigue being identified as the most bothersome symptom with 67% of patients reporting fatigue severity as high [13]. This growing body of evidence highlights the significant impact of fatigue on SCD patients, underscoring the need for further investigation into this critical symptom.

Paradoxically, neither French nor American SCD treatment guidelines address fatigue management [14, 15]. This omission may be linked to the absence of a disease-specific, evidence-based assessment tools, as highlighted by Poku and Pilnick [16] in their study on adolescent fatigue. While various generic fatigue measures are currently used in children, adolescents, and adults with SCD, a specific tool tailored to SCD is still lacking. The multifaceted nature of fatigue, along with its nonspecific presentation and subjective experience, presents a significant challenge for assessment in various medical conditions, including SCD [17–21]. These general difficulties are further compounded in SCD due to the lack of a standardised approach on fatigue terminology, measurement, and the specific dimension it encompasses [17].

Defining the specific aspects of fatigue, or its dimensions, is crucial before its assessment [22]. Billones et al.’s [17] scoping review identified eight dimensions of fatigue measured in various non-oncological medical conditions: physical, cognitive, mental, central, peripheral, emotional, motivational, and psychosocial. Notably, while many researchers agree on the multidimensional nature of fatigue, some advocate for unidimensional assessment (e.g. assessing only the severity of fatigue) in chronic disease [23]. The optimal approach, unidimensional or multidimensional, likely depends on the specific disease and research goals [17]. Critically, the rationale behind the chosen approach and the specific fatigue dimensions explored in SCD studies remain largely unexplored.

A critical gap exists in our understanding of fatigue in SCD, from its assessment to its prevalence. While Eleni et al. (2018) reviewed fatigue measurement in haemoglobinopathies, including but not specifically focused on SCD, their analysis predates the last six years of research and does not explore fatigue dimensions. To address this gap, we conducted a systematic review (1) to identify the psychometric measures of fatigue used in SCD over the past 14 years, (2) to determine the dimensions of fatigue assessed in SCD, and (3) to report levels or prevalence or intensity of fatigue and its correlates in SCD.

## Methods

### Eligibility criteria

#### Inclusion criteria

Studies were included in this systematic review if they met the following criteria:

- Publication format: Peer-reviewed journal articles indexed in major scientific databases (e.g.,PubMed, PsychArticle, PsychInfo).
- Participants: Individuals diagnosed with SCD—children, adolescents, or adults.
- Publication date: Published between January 1st, 2010, and March 31, 2024.
- Comorbidities: Studies with SCD patients with additional psychiatric or medical comorbidities were considered only if the comorbidity was not a specific inclusion criterion. Studies specifically involving patients with a major depressive episode diagnosis were excluded due to potential confounding of fatigue (asthenia) with depressive symptoms.
- SCD Genotypes: All SCD genotypes were included.
- Study type: Studies involving non-human subjects were excluded.
- Language: English and French language studies were included.
- Fatigue Assessment: Studies must have evaluated fatigue using a self-reported psychometric measure.

#### Exclusion criteria

Studies were excluded if they did not meet the fatigue assessment criteria:

- No measurement of fatigue performed.
- Unreported measure of fatigue (e.g. no information on the tool used to measure fatigue).
- Use of non-psychometric tools for fatigue assessment (e.g. visual analogue scale – VAS – or narrative evaluation).
- Fatigue assessment for a single participant (case report).

### Search strategy

A systematic search for relevant articles was conducted across six electronic databases: PubMed, PsycInfo/PsycArticles, Psychology and Behavioral Sciences Collection, Web of Science, Google Scholar. The search strategy combined the following terms: [sickle cell], [fatigue]. Boolean operator ‘AND’ was used to ensure both terms appeared in the retrieved articles either in the title or the abstract. Covidence software was utilised to manage the retrieved article and ensure adherence to Preferred Reporting Items for Systematic Reviews and Meta-Analyses (PRISMA) recommendation [24, 25].

### Study selection

**Fig. 1:**
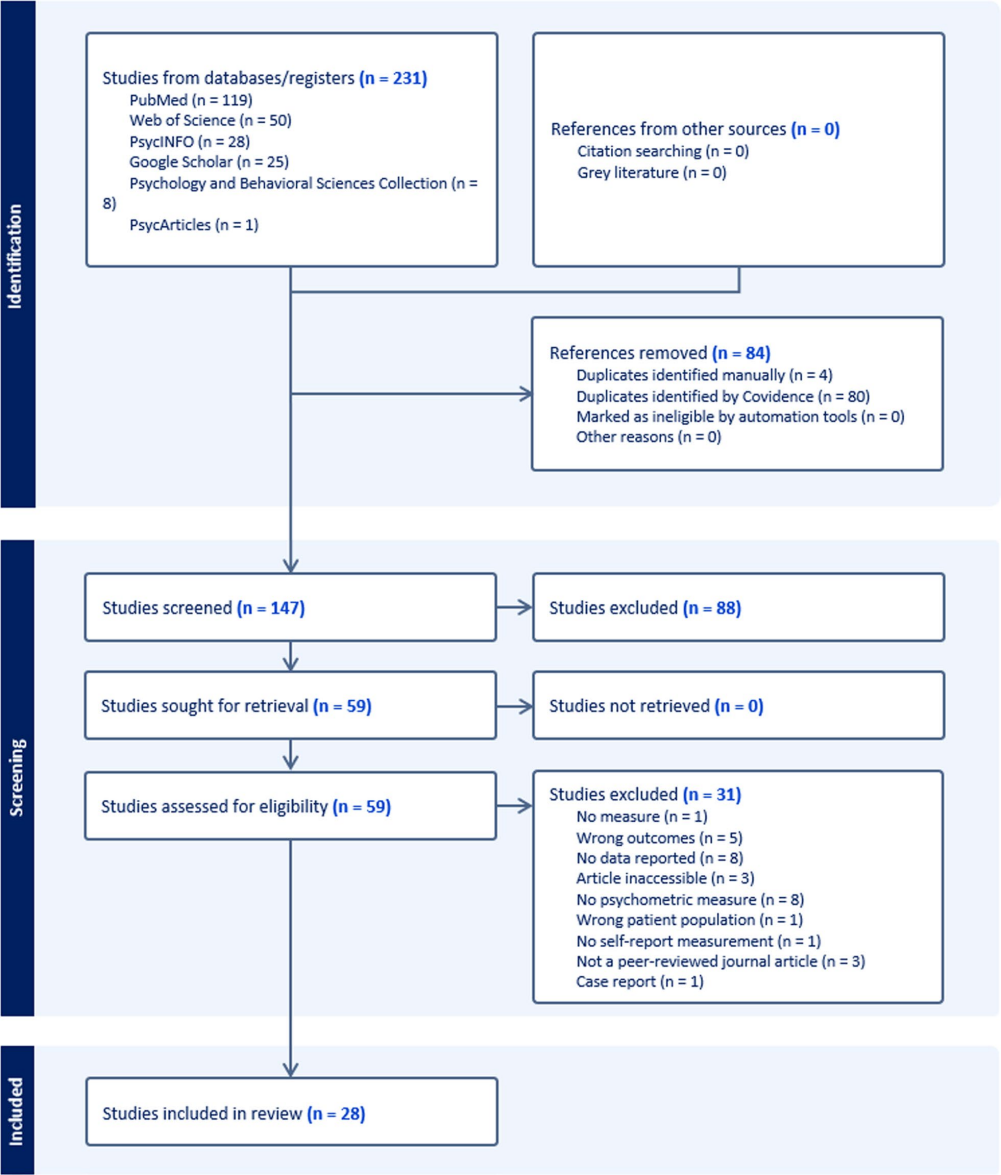
PRISMA flow chart of retrieved sources and screening process (extracted from Covidence)

Two independent reviewers (DOD and AG) screened the retrieved article at each stage of the selection process (except data extraction, which was performed by a single reviewer). Reviewers independently assessed each article for inclusion and exclusion criteria. Any uncertainty or disagreement regarding article inclusion or exclusion was resolved through discussion and consensus between the two reviewers. A flow chart summarising the selection process based on the PRISMA guidelines is depicted in Fig. 1.

### PRISMA compliance and data extraction

This systematic review adheres to the Preferred Reporting Items for Systematic Reviews and Meta-Analyses (PRISMA) guidelines [24, 25]. We used Covidence software to assist in the completion of the various stages of this systematic review. A three-step selection process, starting on April 15, 2024, was employed to identify relevant studies:

Step 1: Initial screening. Articles were initially selected based on titles and abstract retrieval using the search strategy outline above.

Step 2: Eligibility assessment. Full texts of articles identified in step 1 were assessed for eligibility against the following inclusion criteria:

- Participants: Human studies involving children, adolescents, or adults.
- Condition: Studies investigating sickle cell disease (SCD).
- Study type: Peer-reviewed journal articles only.
- Fatigue measurement: Studies utilising self-reported psychometric measures for fatigue assessment.
- Publication date: Published between January 1st, 2010 and,March 31, 2024

Step 3: Data extraction. From studies meeting the inclusion criteria, the following data were extracted and recorded in an Excel spreadsheet: Author(s); Year of publication; Country; Study design; Sample size (for self-report studies only); Participant demographics (gender distribution, age); Study context; SCD genotypes; Fatigue measure name; Dimensions of fatigue assessed; Reported fatigue scores; Type of outcomes; Investigated correlates of fatigue (if any).

To better understand fatigue in SCD, we catalogued the reported correlates of fatigue from all included articles. After a thorough extraction process, we inductively grouped these correlates into nine primary categories. This categorization emerged from a thematic analysis of the identified elements, allowing natural groupings to form rather than relying on predefined criteria. These categories represent the most frequently reported and conceptually distinct correlates found in the literature:

1. Medical/biological outcomes: this category encompasses clinical and laboratory findings, such as the presence of comorbidities (e.g. asthma, avascular necrosis of hips/shoulders, priapism, stroke, hip or joint problems, renal disease, splenic disease, bone and joint disease, heart disease), biological markers (e.g. haemoglobin levels, cytokines, mean corpuscular volume, foetal haemoglobin), treatment status (e.g. hydroxyurea/hydroxycarbamide use (HU), blood transfusion, vitamin D supplementation).
2. Healthcare utilisation which includes hospital admissions and emergency room visits.
3. Pain and related correlates. This category includes the presence and characteristics of pain, as well as factors potentially associated with pain.
4. Sociodemographic outcomes (e.g. age, gender, educational level/attainment, marital status).
5. Psychological and social outcomes which include mental health symptoms (e.g. depressive symptoms, anxiety, stress), social functioning, and internalising symptoms.
6. Quality of life or health-related quality of life (QOL or HRQOL) domains.
7. Disease-related correlates. This category includes disease severity, specific genotype associated with fatigue, and the perceived intrusiveness of the illness on daily life.
8. Neurocognitive outcomes (i.e. working memory, executive functioning, processing speed, cognitive function, intelligence quotient, neurodevelopmental outcomes).
9. Other correlates. This category includes miscellaneous factors not readily classified in the previous categories, such as school or work absenteeism, or adherence to treatment regimen (including barriers to hydroxyurea/hydroxycarbamide use).

This categorisation framework provides a comprehensive structure for analysing the diverse factors associated with fatigue in SCD patients.

For articles with missing or unpublished data points necessary for this analysis (e.g. sample size [N], population characteristics including gender distribution and mean age, and type of fatigue assessment tool), the corresponding authors were contacted via email and requested to provide the missing information in a standardised table format. A follow-up email was sent three weeks after the initial contact if no response was obtained.

Registration process: Our initial submission of this systematic review protocol to PROSPERO on April 23, 2024, encountered a delay due to prioritisation of COVID-19 registrations during the 2020 pandemic.

## Results

### Selection process

The search of the six databases yielded a total of 231 articles (Fig. 1). After removing duplicates, 147 unique studies remained. Title and abstract screening resulted in the exclusion of 88 articles that did not meet our first inclusion criterion of performing a fatigue measurement. This stage also excluded 7 articles (out of 88) mistakenly identified in the databases that did not include the keywords fatigue and sickle cell.

The remaining 59 full-text articles were read in full and assessed for eligibility according to the five inclusion criteria listed above. The reasons for the exclusion of 31 papers, detailed in the flow chart (Fig. 1), are as follows:

- Eight articles did not include psychometric measures of fatigue.
- Eight articles did not specify the reported fatigue data.
- Five articles stated outcomes which did not match the measurements that were made (i.e. ‘wrong outcomes’).
- Three articles’ full text were not accessible. Only their abstract and main results were accessible. They could be processed according to PRISMA standards, however excluded from the review.
- Three articles were not a peer-reviewed journal article.
- One article did not include a fatigue measurement.
- One study did not involve a self-report measure of fatigue.
- One study did not involve the patient population of interest (e.g. sickle-cell patients).
- One study involving a single participant (case report).

After these exclusions, a total of 28 articles reported identifiable data on fatigue measurement in SCD. Table 1 summarised the data extracted from the selected articles (see Table 5 for the reported extracted fatigue scores).

**Table 1:** Prevalence of fatigue assessment tools by age group in 28 selected studies

Children			Adolescents and young adults (< 30 years old)		Adults	
*Name*		*N*	*Name*	*N*	*Name*	*N*
PedsQL™ MFS		6	PedsQL™ MFS	5	FSS	2
Combination of PROMIS Pediatric Fatigue Full Bank and PROMIS Pediatric Fatigue SF*		2	PROMIS CAT Fatigue Domain—Pediatric version and Adult version*	4	PROMIS Adult Fatigue Domain Full Bank*	1
PROMIS Pediatric Fatigue measure*		1	PROMIS Fatigue SF 7-items	4	PROMIS Fatigue SF 6-items	1
PROMIS CAT Fatigue Domain — Pediatric version		1	Combination of PROMIS Pediatric Fatigue Full Bank and PROMIS Pediatric Fatigue SF*	2	PROMIS Fatigue SF 7-items	1
The 4-item SF Fatigue from PROMIS v1.1 Pediatric Profile 25 items*		1	MFSI-SF	2	MFI-20	1
PROMIS Pediatric Fatigue SF		1	BFI	2		
			PROMIS Pediatric Fatigue measure*	1	The Fatigue Severity Subscale of the Brief Fatigue Inventory (BFI)—3 items*	1
			PROMIS Pediatric Fatigue SF	1	A single item from the PROMIS Adult Fatigue item bank (‘I felt tired’) *	1
			FSS	1		
			MFI-20	1		

**N**: number of occurrences in the 28 articles identified

PROMIS: Patient-Reported Outcomes Measurement Information System; SF: Short Form; CAT: Computerised Adaptive Testing; BFI: Brief Fatigue Inventory; FSS: Fatigue Severity Scale; MFI: Multidimensional Fatigue Inventory, MFSI-SF: Multidimensional Fatigue Symptom Inventory – Short Form, PedsQL™ MFS: Pediatric Quality of Life Multidimensional Fatigue Scale

^*^Unclassifiable tools: Tools that did not conform to the established classification criteria due to partial usage, incomplete specification, or a hybrid nature. Please consult the appendix for a detailed discussion of the methodological limitations related to these tools

### Characteristics of the final twenty-eight included studies

Of the 28 selected article, the majority (82%, n = 23) were cross-sectional studies, with longitudinal studies comprising the remaining 18% (n = 5). Three articles [11, 20, 26] originated from the same project database conducted as part of the P30 Centre of Excellence for Biobehavioural Approaches to Symptom Management. Other articles rely on the same participant sample, either as part of a tool validation process [27, 28] or as part of various analyses conducted by the same principal investigator [29–32].

Geographically, most studies were conducted in the USA (n = 23), with two multicentre studies spanning the USA and Egypt, two from Iran, and one from the Netherlands.

The Sample sizes ranged from n = 19 to n = 2201 participants (total N = 5654), encompassing paediatric, adolescent, and adult SCD patients. Participants were recruited from diverse healthcare settings, including routine care, scheduled clinic visits, hospitalisation, and emergency departments during pain crises.

The total sample comprised patients with varying levels of SCD severity, symptoms and treatments due to inconsistencies in reporting participant characteristics across studies (except for gender), calculating overall means for age and SCD genotypes was not feasible. However, based on comparable data, 54.4% of participants were female (n = 28 studies, excluding studies involving the same sample of participants), with a mean age of 15.8 years and a range of 4–45 years (n = 16 studies, excluding studies with the same sample). The most common SCD genotype was SS (72.6%, n = 15 studies), although SC, Sβ^0^ thalassaemia, and Sβ^+^ thalassaemia genotypes were also represented in most studies, albeit to a lesser extent.

The importance attributed to fatigue assessment varied across studies, ranging from a primary outcome measure to a secondary outcome measure. A quarter of the study (25%, n = 7 of 28) prioritised fatigue as their primary outcome measure, while 43% (n = 12 of 28) assessed fatigue as a secondary outcome. The remaining 32% (n = 9 of 28) focused on the development, testing, validation, or comparison of measuring instruments. Of these instruments-focused studies, only two specifically concerned fatigue assessment tool [26, 33], while the remaining seven studies utilised generic tools to assess fatigue alongside other domains.

### Overall picture of the identified fatigue measurement tools

#### General description of the 16 fatigue assessment tool identified

From January 1st, 2010, to March 31, 2024, a total of 16 different fatigue instruments were used with children (up to 17 years old), adolescents and young adults (AYA, under 30 years old), and adults suffering from SCD. Ten of these instruments or versions were designed for adults but six were also used with adolescents or young adults. Six instruments or versions were intended for children but five were also used in some studies involving adolescents and young adults.

Table 2 classified the instruments identified among the three populations with their frequency of use. Notably since each study included one to three psychometric tools, and potentially surveyed multiple age groups, the number of occurrences was not equal to the number of eligible articles.

**Table 2:** Overview of fatigue assessment in sickle cell disease: a synthesis of 28 studies

Author(s), Year	Country	Study design	Population					Study context	Fatigue Measure(s)	Investigated correlates of fatigue	Type of outcomes
			*Type*	*Age*	Sample size*	*Sex (female, %)*	*SCD genotypes*				
[54]	USA	Cross-sectional	Children/Adolescents	Range: 5–18/Mean age (SD): 11.3 (3.8)	1393	46	SS or Sβ0 thalassaemia: 68.3%, SC or Sβ + thalassaemia: 31.7%	Patients who have to return to their clinics for routine care	PedsQL™ MFS (version 4.0)	Pain Age/Gender, Phenotypes groups, Medical outcomes, No. of days children missed school/caregivers missed work	Fatigue as secondary outcome measure
[33]	USA	Cross-sectional	Children/Adolescents	Range: 5–18/Mean age (SD) (n = 319 families): 9.70 (4.83)	240 paediatric patients/319 families	52 (for n = 319 families)	Any genotype	Patients and/or parents who presented for a clinic visit	PedsQL™ MFS	Disease severity, HRQOL, Medical outcome	Instrument development, test, validation or comparison (specific tool for fatigue)
[11]	USA	Cross-sectional	Adolescents/Young adults	Range: 15–30/Mean age: (SD) 22.5 (4.1)	60	60	SS: 65%, SC: 18.4%, Sβ0 thalassaemia: 3.3%, Sβ + thalassaemia: 10%, Unknow: 3.3%	Patients with mild or severe SCD, asked to complete a survey packet during a hospitalisation or clinic visit	PROMIS Fatigue SF 7-items / MFSI-SF/BFI	Pain, Sleep quality, Anxiety, Depression, Perceived stress Medical outcome, Age/Gender Disease severity, Quality of life	Fatigue as primary outcome measure
[20]	USA	Cross-sectional	Adolescents / Young adults	Range: 15–30/Mean age: 22.5	60	60	-	Patients initially included in the Ameringer et al. (2014) study	PROMIS Fatigue SF 7-items	Depressive symptoms, Perceived stress, Medical outcome	Fatigue as primary outcome measure
[55]	Netherlands	Cross-sectional	Children	Range: 8–16/Mean age (SD): 12.5 (2.7)	38	42	Homozygous SCD: 95%, Sβ0 thalassaemia: 5%	Patients recruited through regular hospital visits or by letter, with severe SCD, clinically stable during study visit (i.e. no infection or crisis for > 4 weeks prior to the visit)	PedsQL™ MFS	Volume of cerebral white matter hyperintensities	Fatigue as secondary outcome measure
[28]	USA	Cross-sectional	Children/Adolescents	Range: 8–17/Mean age (SD): 12.49 (2.82)	235 (234 for PROMIS)	49.8	SS: 76.5%, SC: 16.7%, Sβ + thalassaemia: 4.7%, Sβ0 thalassaemia: 1.3%	Patients recruited at clinic visits for routine care, HU monitoring, or chronic transfusions	Combination of PROMIS Pediatric Fatigue Full Bank (36 items) and PROMIS Pediatric Fatigue SF (10 items)	Pain/need for treatment of pain	Instrument development, test, validation or comparison (generic tool for assessing fatigue among other domains)
[56]	Iran	Longitudinal	Adults	Over 18, Mean age (SD): 25.98 (7.18)	53 (final sample)	67.9	SS: 64.2%, Sickle beta thalassaemia: 35.8%	Patients recruited in their clinic to follow a self-management program targeting pain, fatigue, depression, anxiety and stress	FSS	Depression, Anxiety, Stress, Pain (frequency and duration)	Fatigue as secondary outcome measure
[12]	USA	Cross-sectional	Children	Range: 8–16/Mean age: 12.34	32	46.9	SS: 81.8%, SC: 12.1%, Sβ0: 3.0%^a^	Patients and a primary caregiver recruited from a Paediatric Sickle Cell Clinic and approached during their regularly scheduled haematology clinic visits	PedsQL™ MFS	HRQOL, Working memory, Executive functioning, Metacognition, Internalising symptoms, Pain, Hospitalisation, SCD diagnosis, Sex, Medical outcomes	Fatigue as primary outcome measure
[57]	USA	Longitudinal	Children/Adolescents	Range: 8–17/Mean age (SD): 12.5 (3.1)	121	56.2	SS or Sβ0 thalassaemia: 73.6%, SC or Sβ + thalassaemia: 24.8%, Other: 1.6%	Patients with acute care visits for pain in the previous year, who completed questionnaires at an initial routine healthcare encounter in the sickle cell clinic and/or at the end of hospitalisation for pain	PROMIS Pediatric Fatigue measure	Pain hospitalisation	Instrument development, test, validation or comparison (generic tool for assessing fatigue among other domains)
[27]	USA	Cross-sectional	Children/Adolescents	Range: 8–17/Mean age (SD): 12.5 (2.8)	235 (234 for PROMIS)	49.8	SS or Sβ0 thalassaemia: 77.5%, SC or Sβ + thalassaemia: 21.3%, Other: 0.9%, Unknown: 0.4%	Patients recruited at the time of routine clinic visits, likely to be relatively asymptomatic or presenting to the clinic for monthly HU (patients followed by two large Sickle Cell programs and initially included in the DeWalt et al. 2015) study)	Combination of PROMIS Pediatric Fatigue Full Bank and PROMIS Pediatric Fatigue SF	Gender/Age, Pain, Hospitalisation, Emergency department visits for pain, Home-managed pain episodes, Medical outcomes, SCD genotypes	Instrument development, test, validation or comparison (generic tool for assessing fatigue among other domains)
[26]	USA	Cross-sectional	Adolescents/Young adults	Range: 15–30/Mean age (SD): 22.5 (4.1)	60	60		Patients with SCD included in the study by Ameringer et al. (2014)	PROMIS Fatigue SF 7-items / MFSI-SF/BFI	Stress, Depressive symptoms	Instrument development, test, validation or comparison (specific tool for fatigue)
[31]	USA/Egypt	Cross-sectional	Adolescents / Young adults	Range: 12–22/Median age (IQR): 13.5 (12–18)	34	41	SS: 85.3%, SC: 8.8%, Sβ0:5.9%	Patients treated with HU and who were recruited at their scheduled appointment at the comprehensive outpatient sickle cell clinic or HU clinic	PROMIS CAT Fatigue Domain—Pediatric version and Adult version	Adherence to HU (MMAS-8), Medical outcomes	Fatigue as secondary outcome measure
[32]	USA/Egypt	Cross-sectional	Adolescents/Young adults	Range: 12–22/Median age (IQR): 13.5 (12–18)	34	41	SS: 85.3%, SC: 8.8%, Sβ0:5.9%	Patients treated with HU and who were recruited at their scheduled appointment at the comprehensive outpatient sickle cell clinic or HU clinic	PROMIS CAT Fatigue Domain—Pediatric version and Adult version	Barriers to HU adherence (recall barriers, negative beliefs, access)	Fatigue as secondary outcome measure
[58]	USA	Longitudinal	Children/Adolescents	Range: 4–21/Mean age (SD): 13.5 (4.5)	187	50	SS: 93%, Sβ0 thalassaemia: 7%	Patients who presented to the emergency department (ED) with a painful vaso-occlusive crisis	PedsQL™ MFS	Pain	Instrument development, test, validation or comparison(generic tool for assessing fatigue among other domains)
[59]	USA	Cross-sectional	Children/Adolescents	Range: 7–18, Mean age (SD): 12.1 (3.4)	19 (18 for PedsQL™ MFS)	63.2	SS: 52.6%, SC: 21.1%, Sβ thalassaemia: 26.4%	Participants were recruited from a tertiary care SCD clinic; referred to neurodevelopmental testing because of academic or behavioural difficulties, and all had undergone a neurodevelopmental evaluation	PedsQL™ MFS	Sleep measures and neurodevelopmental outcomes	Fatigue as primary outcome measure
[60]	USA	Cross-sectional	Adults	18–55 and over	490	64	SS: 65%, SC: 20%, Sβ + or Sβ0 thalassaemia: 10%, Type unspecified: 5%	Patients seen in US outpatient clinics, in stable condition and/or under treatment who differed in their SCD severity	PROMIS Fatigue SF 7-items	SCD severity	Instrument development, test, validation or comparison (generic tool for assessing fatigue among other domains)
[9]	Iran	Cross-sectional	Adolescents / Adults	16 and older	97	64.9	SS: 64.9%, Sickle Beta Thalassaemia: 35.1%	Patients who come for a routine clinic visit or hospitalisation	FSS	Depression, Anxiety, Stress, Medical outcomes Age, sex, Marital status, Educational level, SCD genotype	Fatigue as primary outcome measure
[40]	USA	Cross-sectional	Adults	Median age (IQR): 35 (23–41)	47	79	SS: 57.5%, SC: 19.2%, Sβ0 thalassaemia: 6.4%, Sβ + thalassaemia: 8,5%, Other/not sure: 8,5%	Patients recruited through advertisements, booths at SCD conferences and from SCD clinics; who were considering a disease-modifying treatment (HU, bone marrow transplantation, or chronic blood transfusion), and had not obtained the treatment option of interest in the past 12 months	PROMIS Fatigue SF 6-items	Gender, Pain, Age	Fatigue as secondary outcome measure
[29]	USA	Cross-sectional	Adolescents/Young adults	Range: 12–22/Mean age (SD): 14.8 (2.9)	34	41	SS: 85.3%, SC: 8.8%, Sβ0:5.9%	Patients treated with HU and who were recruited at their scheduled appointment at the comprehensive outpatient sickle cell clinic or HU clinic	PROMIS CAT Fatigue Domain—Pediatric version and Adult version	Age, Gender, Chronic pain, QOL (PROMIS domains)	Fatigue as secondary outcome measure
[30]	USA	Cross-sectional	Adolescents/Young adults	12 and older/Median age (IQR): 14 (12–18)	34	41	SS: 85%	Patients treated with HU and who were recruited at their scheduled appointment at the comprehensive outpatient sickle cell clinic or HU clinic	PROMIS CAT Fatigue Domain—Pediatric version and Adult version	Hospitalisations, Emergency room visits, Inpatient hospital length of stay	Fatigue as secondary outcome measure
[61]	USA	Cross-sectional	Young adults	Range: 18–24/Mean age (SD): 20.81 (1.73)	45	53	SS: 69%, SC: 27%, Sβ + thalassaemia: 4%	Patients receiving care in a tertiary children’s hospital or affiliated adult hospital in the Midwestern United States	PROMIS Fatigue SF 7-items	Quality of life (PedsQL), Disease severity, Medical outcomes	Instrument development, test, validation or comparison (generic tool for assessing fatigue among other domains)
[41]	USA	Cross-sectional	Adults	Range: 18–45/Mean age (SD): 29.2 (7.2)	2201	57.6	Genotypes reported for n = 2196^a^: SS or Sβ0:73.1%, SC: 20.5%, Sβ + : 5.1%, Other (Hb S/HPFH, SE, SO, SD): 1%	Participants enrolled in the SCD Implementation Consortium registry; recruited from SCD clinics, inpatient units, emergency departments, pain centres, community events and via targeted phone calls	A single item from the PROMIS Adult Fatigue item bank (‘I felt tired’)	Sex, Educational attainment, Sleep impact, Social functioning, Depressive symptoms, Cognitive function	Fatigue as secondary outcome measure
[62]	USA	Longitudinal	Children/Adolescents	Range: 5–20/Mean age (SD): 11 (4)	21	57	SS: 100%	Children recruited from the Comprehensive Sickle Cell Center at the Children’s Hospital of Philadelphia and for a vitamin D supplementation study	PROMIS Pediatric Fatigue SF	Medical outcome (vitamin D supplementation)	Fatigue as secondary outcome measure
[63]	USA	Cross-sectional	Children	Range: 8–17/Mean age (SD): 13.2 (3.1)	90	48.9	SS: 55.6%, SC: 16.7%, Sβ0 thalassaemia: 1.1%, Sβ + thalassaemia: 1.1%, Other: 2.2%	Children with SCD without disease exacerbation, recruited from a haematology clinic	The 4-item SF Fatigue from PROMIS v1.1 Pediatric Profile 25 items / PROMIS CAT Fatigue Domain -Pediatric version		Instrument development, test, validation or comparison (generic tool for assessing fatigue among other domains)
[64]	USA	Cross-sectional	Adults	Range: 19–58/Median age (SD): 33.4 (10.0)	33	45.5	SS: 63.6%, SC: 30.3%, Sβ + : 6.1%	Patients with or without chronic pain, presented to the Adult SCD Clinic for care, hospitalisation for pain or when they accompanied other family members to appointments	PROMIS Adult Fatigue Domain Full Bank	Chronic pain	Fatigue assecondary outcome measure
[65]	USA	Longitudinal	Adolescents / Young adults	Range: 16–25/Mean age (SD): 19 (3.3)	106	53.8	SS or Sβ0 thalassaemia: 53,8%, SC or Sß + thalassaemia or other: 46,2%	Patients included in the Sickle Cell Clinical Research Intervention Program (SCCRIP), a longitudinal lifetime cohort study	PedsQL™ MFS	Intelligence quotient, working memory, processing speed (measures of neurocognitive performance)	Fatigue as primary outcome measure
[10]	USA	Cross-sectional	Adults	Range: 18–69	60	69.5	SS or Sβ0:64.4%	Patients treated or not with HU, recruited from community organisations and academic medical centres	The Fatigue Severity Subscale of the Brief Fatigue Inventory (BFI)—3 items	Illness intrusiveness, Pain severity, Depressive symptoms, Anxiety symptoms, HRQOL	Fatigue as primary outcome measure
[66]	USA	Cross-sectional	Adolescents/Adults	Range: 14–73	52	51.9	SS or Sβ0:63.5%, SC: 26.9%, Sβ + thalassaemia: 9.6%	Patients diagnosed with traditional Chinese medicine (TCM) and experiencing chronic pain in the past 6 months or at least one VOC in the past 12 months	MFI		Fatigue as secondary outcome measure

Note. *self-report only; ^**a**^ problem in reported statistics

**PedsQL™ MFS**: Pediatric Quality of Life Multidimensional Fatigue Scale, **PROMIS**: Patient-Reported Outcomes Measurement Information System; **SF**: Short Form; **CAT**: Computerised Adaptive Testing; **BFI**: Brief Fatigue Inventory; **FSS**: Fatigue Severity Scale; **MFI**: Multidimensional Fatigue Inventory, **MFSI-SF**: Multidimensional Fatigue Symptom Inventory – Short Form, **HRQOL**: Health-related quality of life, **HU**: hydroxyurea/hydroxycarbamide

**SD**: standard deviation, **SE**: standard error, **IQR**: interquartile range

The most frequently employed instrument for fatigue measurement was the Patient-Reported Outcomes Measurement Information System (n = 17; PROMIS;,34). Developed by the National Institutes of Health (NIH), PROMIS is a person-centred system design to assess various symptoms and health-related quality of life (HRQOL) domains. For fatigue specifically, PROMIS offers several formats applicable to both children/adolescents and adults. Seventeen studies (60.7%) utilised PROMIS versions including:

- Paediatric version (n = 10). The PROMIS Pediatric Fatigue Short-Form was used in one study, and the PROMIS CAT (Computerised Adaptive Testing) Fatigue Domain—Pediatric version in another. While several other tools had paediatric versions, their classification was challenging due to their hybrid nature (combination tools), unspecified versions, or partial usage (e.g., only a few items). For instance, combinations of the PROMIS Pediatric Fatigue Full Bank and Short-Form were used in two studies, the undifferentiated PROMIS CAT Fatigue Domain—Pediatric and Adult versions in four studies, an unspecified PROMIS Pediatric Fatigue measure in one study, and four fatigue items Short-Form from the PROMIS v1.1 Pediatric Profile 25 measure in one study.
- Adult version (n = 12). PROMIS Adult Fatigue Domain Full Bank was used in one study, the PROMIS Fatigue Short Form 7 items in five studies and the PROMIS Fatigue Short Form 6 items in one study. Similar to the paediatric versions, some of the adult tools were not easily classifiable. For example, one study use of a single item from the PROMIS Adult Fatigue item bank, and four studies utilised the undifferentiated paediatric and adult versions of the PROMIS CAT Fatigue Domain, as mentioned above.

The second most common employed tool for assessing fatigue in children and young adults was the Pediatric Quality of Life Inventory Multidimensional Fatigue Scale (PedsQL™ MFS;,35). It was utilised in 7 studies (25%).

Four additional tools were used in adolescent, young adult and adult populations:

- N = 3 studies (10.7%) used the Brief Fatigue Inventory (BFI; 36), but one of these used it partially, exploiting only the Fatigue Severity Subscale.
- N = 2 studies (7.1%) used the Fatigue Severity Scale (FSS; 37).
- N = 2 studies (7.1%) used the Multidimensional Fatigue Symptom Inventory – Short Form (MFSI-SF; 38).
- N = 1 study (3.6%) used the Multidimensional Fatigue Inventory (MFI or MFI-20;,39).

#### Unidimensional versus multidimensional scales

The fatigue instruments identified in this review were categorised based on their dimensionality. Unidimensional fatigue scales assess fatigue on a single dimension, such as overall fatigue (e.g. PROMIS), fatigue severity (e.g. BFI) or fatigue impact (e.g. FSS). Thirteen instruments were classified as unidimensional scales including the Brief Fatigue Inventory (BFI), Fatigue Severity Scale (FSS), and all variants of the PROMIS listed above. Among these instruments, six are challenging to classify due to partial usage, incomplete specification, or a hybrid nature; however, they still pertain to a unidimensional measure.

In contrast, three instruments were categorised as multidimensional scales: the Multidimensional Fatigue Inventory (MFI-20), the Multidimensional Fatigue Symptom Inventory-Short Form (MFSI-SF), and the Pediatric Quality of Life Inventory Multidimensional Fatigue Scale (PedsQL™ MFS). Multidimensional fatigue scales capture various aspects of fatigue including general fatigue, physical fatigue, mental fatigue, cognitive fatigue, emotional fatigue, impact of fatigue on motivation and activity, energy levels (vigour) and sleep/rest fatigue.

Table 3 provides a comprehensive overview of the 16 identified fatigue instruments.

**Table 3:** Comparative analysis of fatigue assessment tools: dimensions, reliability, frequency, and references

Unidimensional instruments				
Name	Dimension assessed	Internal consistency (reference)	Nb of occurrences	Study ID
PROMIS Pediatric SF Fatigue Domain	Overall fatigue	0.80 (68)	1	[15]
PROMIS CAT Fatigue Domain—Pediatric version	Overall fatigue	Not found	1	[20]
PROMIS Adult Fatigue Domain Full Bank	Overall fatigue	> 0.91 (34)	1	[22]
PROMIS Fatigue SF — 7 items	Overall fatigue	> 0.90 (69)	5	[3, 4, 16, 17, 19]
PROMIS Fatigue SF — 6 items	Overall fatigue	0.93 (70)	1	[10]
BFI	Fatigue severity	0.96 (36)	2	[3, 4]
FSS	Fatigue impact	0.88 (37)	2	[1, 2]

Unclassifiable tools* referring to unidimensional instruments				
Name	Dimension assessed	Internal consistency (reference)	Nb of occurrences	Study ID
Combination of PROMIS Pediatric Fatigue Full Bank and PROMIS Pediatric Fatigue SF	Overall fatigue	0.87 for Full Bank (71) 0.80 for SF (68)	2	[11, 14]
PROMIS Pediatric Fatigue measure	Overall fatigue		1	[12]
The 4-item SF Fatigue from PROMIS v1.1 Pediatric Profile 25 items	Overall fatigue	‘High internal consistency’ for the complete tool (72)	1	[20]
PROMIS CAT Fatigue Domain—Pediatric and Adult versions	Overall fatigue	Not found for Pediatric version > 0.90 (69) for Adult version	4	[6–9]
A single item from the PROMIS Adult Fatigue item bank			1	[18]
The Fatigue Severity Subscale of the BFI—3 items	Fatigue severity	0.96 for the complete tool (36)	1	[21]

Multidimensional instruments				
*Name*	*Dimension assessed*	*Internal consistency (reference)*	*Nb of occurrences*	*Study ID*
MFI or MFI-20	1) General fatigue 2) Physical fatigue 3) Mental fatigue 4) Reduced motivation 5) Reduced activity	0.72 to 0.86 (73)	1	[28]
MFSI-SF	1) General fatigue 2) Physical fatigue 3) Mental fatigue 4) Emotional fatigue 5) Vigor	0.87 to 0.96 (38)	2	[3, 4]
PedsQL™ MFS	1) General fatigue 2) Sleep/rest fatigue 3) Cognitive fatigue	0.77 to 0.90 for patient self-report 0.90 to 0.97 for parent report (33)	7	[5, 13, 23–27]

**PROMIS**: Patient-Reported Outcomes Measurement Information System; **SF**: Short Form; **CAT**: Computerised Adaptive Testing; **BFI**: Brief Fatigue Inventory; **FSS**: Fatigue Severity Scale; **MFI**: Multidimensional Fatigue Inventory, **MFSI-SF**: Multidimensional Fatigue Symptom Inventory – Short Form, **PedsQL™ MFS**: Pediatric Quality of Life Multidimensional Fatigue Scale

*Unclassifiable tools: Tools that did not conform to the established classification criteria due to partial usage, incomplete specification, or a hybrid nature. Please consult the appendix for a detailed discussion of the methodological limitations related to these tools.

### Consequences in use: comparability of outcomes obtained

Shared and divergent dimensions. Three multidimensional fatigue instruments (MFI-20, MFSI-SF, PedsQL MFS) employed in the final 28 articles assess fatigue through various dimensions, although some overlap exists. All three tools incorporate ‘general fatigue’ as a dimension. Additionally, both MFI-20 and MFSI-SF assess ‘physical fatigue’ and ‘mental fatigue’. While the definition of these shared dimensions appears conceptually similar across instruments, full definitions are not always explicitly provided. The PedsQL™ MFS, for instance, utilises examples to illustrate the concepts.

Distinguishing between ‘mental fatigue’ and ‘cognitive fatigue’ based solely on the instrument information proves challenging. This lack of clear differentiation is also evident when compared to the standard definitions cited in Billones et al. [17]. Table 4 summarised the various dimensions of fatigue assessed by the instruments along with their definitions (from the instrument authors) and the standard definitions from Billones et al. [17] as comparison.

**Table 4:** Comparative analysis of fatigue dimensions definition: definition used in multidimensional instruments vs. standard definitions according to Billones et al. (2021)

	General fatigue	Physical fatigue	Mental fatigue	Cognitive fatigue	Motivational fatigue		Emotional fatigue	Reduced activity	Reduced motivation	Vigor	Sleep/rest fatigue
Multidimensional Fatigue Inventory (MFI or MFI-20) [39]	General remarks of a person concerning his or her functioning, for example ‘I feel rested’ (Smets et al., 1995)	Physical sensations related to fatigue/the feeling of tiredness (Lin et al., 2009; Smets et al., 1995)	Cognitive symptoms, such as having difficulty concentrating (Smets et al., 1995)					A frequent, but not necessarily, consequence of fatigue and consequence of reduced motivation on the level of activity (Smets et al., 1995)	Lack of motivation for starting any activity (Lin et al., 2009; Smets et al., 1995)		
Multidimensional Fatigue Symptom Inventory-Short Form (MFSI-SF) [38, 73]	Global experience of fatigue	Somatic symptoms of fatigue	Cognitive symptoms of fatigue			Affective symptoms of fatigue				Behavioral symptoms of fatigue (rationally derived scale)/Measure of patient’s energy level (empirically derived scale)	
Pediatric Quality of Life Multidimensional Fatigue Scale (PedsQL™ MFS) [35, 74]	e.g. ‘I feel tired.’; ‘I feel too tired to do things that I like to do.’			e.g. ‘It is hard for me to keep my attention on things.’; ‘It is hard for me to remember what people tell me.’							e.g. ‘I feel tired when I wake up in the morning.’; ‘I rest a lot.’
Standards definitions in non-oncologic medical conditions [17]		Debilitating physical exhaustion or a distressing lack of energy not relieved by sleep or rest	Mental exhaustion that appears especially during sensory stimulation or following mentally strenuous tasks	A symptom interfering with a person’s ability to carry out cognitive activities and can be persistent and overwhelming, which differs from normal fatigue	A symptom that is disruptive in terms of motivation and in initiating activities	An unpleasant symptom that is strongly associated with depression and is extremely disruptive to health-related quality of life					

Challenges in comparing fatigue scores: Analysis of fatigue severity across studies was hindered by using different instruments and reporting methods. Table 5 summarised the fatigue scores reported by the 28 articles, specifying the type of tool used, the number of items, the method used for interpretation, the threshold score or cut-off of the tool if it exists, as well as the number of respondents, the type of report and the condition according to the fatigue score presented.

**Table 5:** Comparative analysis of reported fatigue scores

PROMIS pediatric fatigue measures							
Reference	Type	Nb of items	Interpretation/Cut-off/Calibration	N total	Report type	Score condition	Score
[11]	Combination of PROMIS Pediatric Fatigue Full Bank and PROMIS Pediatric Fatigue SF*	*	T-score metric (M = 50, SD = 10), Higher scores indicate greater fatigue	234	Mean (SD)	Total sample	46.7 (13.0)
[11]	Combination of PROMIS Pediatric Fatigue Full Bank and PROMIS Pediatric Fatigue SF*	*	T-score metric (M = 50, SD = 10), Higher scores indicate greater fatigue	*	Mean (SD)	8–11 years	44.6 (14.4)
[11]	Combination of PROMIS Pediatric Fatigue Full Bank and PROMIS Pediatric Fatigue SF*	*	T-score metric (M = 50, SD = 10), Higher scores indicate greater fatigue	*	Mean (SD)	12–17 years	48.0 (11.9)
[11]	Combination of PROMIS Pediatric Fatigue Full Bank and PROMIS Pediatric Fatigue SF*	*	T-score metric (M = 50, SD = 10), Higher scores indicate greater fatigue	*	Mean (SD)	Male	44.6 (12.4)
[11]	Combination of PROMIS Pediatric Fatigue Full Bank and PROMIS Pediatric Fatigue SF*	*	T-score metric (M = 50, SD = 10), Higher scores indicate greater fatigue	*	Mean (SD)	Female	48.7 (13.3)
[11]	Combination of PROMIS Pediatric Fatigue Full Bank and PROMIS Pediatric Fatigue SF*	*	T-score metric (M = 50, SD = 10), Higher scores indicate greater fatigue	*	Mean (SD)	SS/SB0 thalassemia	46.6 (12.8)
[11]	Combination of PROMIS Pediatric Fatigue Full Bank and PROMIS Pediatric Fatigue SF*	*	T-score metric (M = 50, SD = 10), Higher scores indicate greater fatigue	*	Mean (SD)	SC/SB + thalassemia	47.1 (14.0)
[11]	Combination of PROMIS Pediatric Fatigue Full Bank and PROMIS Pediatric Fatigue SF*	*	T-score metric (M = 50, SD = 10), Higher scores indicate greater fatigue	*	Mean (SD)	Pain in past 7 days	53.7 (12.2)
[11]	Combination of PROMIS Pediatric Fatigue Full Bank and PROMIS Pediatric Fatigue SF*	*	T-score metric (M = 50, SD = 10), Higher scores indicate greater fatigue	*	Mean (SD)	No pain in past 7 days	43.6 (12.2)
[11]	Combination of PROMIS Pediatric Fatigue Full Bank and PROMIS Pediatric Fatigue SF*	*	T-score metric (M = 50, SD = 10), Higher scores indicate greater fatigue	*	Mean (SD)	Current hip or joint issues	54.5 (13.6)
[11]	Combination of PROMIS Pediatric Fatigue Full Bank and PROMIS Pediatric Fatigue SF*	*	T-score metric (M = 50, SD = 10), Higher scores indicate greater fatigue	*	Mean (SD)	No current hip or joint issues	45.0 (12.4)
[12]	PROMIS Pediatric Fatigue measure*	*	T-score metric (M = 50, SD = 10), Higher scores indicate greater fatigue	91	Mean (SE)	Baseline visit	52.2 (1.5)
[12]	PROMIS Pediatric Fatigue measure*	*	T-score metric (M = 50, SD = 10), Higher scores indicate greater fatigue	80	Mean (SE)	Follow-up visit	53.0 (1.5)
[12]	PROMIS Pediatric Fatigue measure*	*	T-score metric (M = 50, SD = 10), Higher scores indicate greater fatigue	51	Mean (SE)	Hospital episode	61.7 (1.8)
[12]	PROMIS Pediatric Fatigue measure*	*	T-score metric (M = 50, SD = 10), Higher scores indicate greater fatigue	16	Mean (SE)	Recovery episode	50.9 (2.6)
[14]	Combination of PROMIS Pediatric Fatigue Full Bank and PROMIS Pediatric Fatigue SF*	10 (SF)* + 36 (Full bank)	T-score metric (M = 50, SD = 10), Higher scores indicate greater fatigue	72	Mean (SD)	Home treatment for pain in past week	54 (12)
[14]	Combination of PROMIS Pediatric Fatigue Full Bank and PROMIS Pediatric Fatigue SF*	10 (SF)* + 36 (Full bank)	T-score metric (M = 50, SD = 10), Higher scores indicate greater fatigue	162	Mean (SD)	No home treatment for pain in past week	44 (12)
[15]	PROMIS Pediatric Fatigue SF	*	T-score metric (M = 50, SD = 10), Higher scores indicate greater fatigue	21	Mean (SD)	Baseline	51.7 (11.4)
[15]	PROMIS Pediatric Fatigue SF	*	T-score metric (M = 50, SD = 10), Higher scores indicate greater fatigue	21	Mean (SD)	6 weeks vitamin D supplementation	48.6 (10.2)
[15]	PROMIS Pediatric Fatigue SF	*	T-score metric (M = 50, SD = 10), Higher scores indicate greater fatigue	20	Mean (SD)	12 weeks vitamin D supplementation	46.4 (14.0)
[20]	The 4-item SF Fatigue from PROMIS v1.1 Pediatric Profile 25 items	4*	Item Response Theory (IRT) models*	89	Mean (SD)	SF version	45.34 (10.3)
[20]	The 4-item SF Fatigue from PROMIS v1.1 Pediatric Profile 25 items	4*	Item Response Theory (IRT) models*	89	Range of scores (Min–Max)	SF version	35.4–71.9
[20]	PROMIS CAT Fatigue Domain—Pediatric version	Range of items: 5–12, Mean (SD): 10 (3)	Item Response Theory (IRT) models*	88	Mean (SD)	CAT version	40.97 (14.66)
[20]	PROMIS CAT Fatigue Domain—Pediatric version	Range of items: 5–12, Mean (SD): 10 (3)	Item Response Theory (IRT) models*	88	Range of scores (Min–Max)	CAT version	25.6–78.1
[6]	PROMIS CAT Fatigue domain—Pediatric version and Adult version*	Vary within and in-between patients*	T-score metric (M = 50, SD = 10), Higher scores indicate greater fatigue	34*	Median (IQR)	Total sample	49.4 (39.9–60.9)
[6]	PROMIS CAT Fatigue domain—Pediatric version and Adult version*	Vary within and in-between patients*	T-score metric (M = 50, SD = 10), Higher scores indicate greater fatigue	*	Median (IQR)	12–17 years	46.2 (35–57.1)
[6]	PROMIS CAT Fatigue domain—Pediatric version and Adult version*	Vary within and in-between patients*	T-score metric (M = 50, SD = 10), Higher scores indicate greater fatigue	*	Median (IQR)	18–22 years	60.7 (51–62.6)
[6]	PROMIS CAT Fatigue domain—Pediatric version and Adult version*	Vary within and in-between patients*	T-score metric (M = 50, SD = 10), Higher scores indicate greater fatigue	*	Median (IQR)	Male	45.3 (39.1–54.1)
[6]	PROMIS CAT Fatigue domain—Pediatric version and Adult version*	Vary within and in-between patients*	T-score metric (M = 50, SD = 10), Higher scores indicate greater fatigue	*	Median (IQR)	Female	57.1 (48.8–62)
[6]	PROMIS CAT Fatigue domain—Pediatric version and Adult version*	Vary within and in-between patients*	T-score metric (M = 50, SD = 10), Higher scores indicate greater fatigue	*	Median (IQR)	Chronic pain	61.5 (60.9–62)
[6]	PROMIS CAT Fatigue domain—Pediatric version and Adult version*	Vary within and in-between patients*	T-score metric (M = 50, SD = 10), Higher scores indicate greater fatigue	*	Median (IQR)	No chronic pain	49 (39.9–60)
[7]	PROMIS CAT Fatigue domain—Pediatric version and Adult version*	*	T-score metric (M = 50, SD = 10), Higher scores indicate greater fatigue	14	Median (IQR)	No hospitalizations in 1 year prior	45.3 (35.2–49.7)
[7]	PROMIS CAT Fatigue domain—Pediatric version and Adult version*	*	T-score metric (M = 50, SD = 10), Higher scores indicate greater fatigue	11	Median (IQR)	1–3 hospitalizations in 1 year prior	48.8 (35–62.4)
[7]	PROMIS CAT Fatigue domain—Pediatric version and Adult version*	*	T-score metric (M = 50, SD = 10), Higher scores indicate greater fatigue	9	Median (IQR)	Hospitalizations > or = 4 in 1 year prior	61.5 (57.1–63.1)
[8]	PROMIS CAT Fatigue domain—Pediatric version and Adult version*	*	T-score metric (M = 50, SD = 10), Higher scores indicate greater fatigue	31*	Median (IQR)	Total sample	49.4 (39.9–60.9)
[8]	PROMIS CAT Fatigue domain—Pediatric version and Adult version*	*	T-score metric (M = 50, SD = 10), Higher scores indicate greater fatigue	22	Median (IQR)	Low adherence to HU (according to MMAS-8)	55.5 (45.8–62.2)
[8]	PROMIS CAT Fatigue domain—Pediatric version and Adult version*	*	T-score metric (M = 50, SD = 10), Higher scores indicate greater fatigue	22	Median (IQR)	Moderate/high adherence to HU (according to MMAS-8)	39.1 (28.3–43.7)
[8]	PROMIS CAT Fatigue domain—Pediatric version and Adult version*	*	T-score metric (M = 50, SD = 10), Higher scores indicate greater fatigue	12	Median (IQR)	Low HbF % levels < 16%	52.6 (35–62.7)
[8]	PROMIS CAT Fatigue domain—Pediatric version and Adult version*	*	T-score metric (M = 50, SD = 10), Higher scores indicate greater fatigue	14	Median (IQR)	High HbF % levels > or = 16%	47.7 (39.5–58.8)
[8]	PROMIS CAT Fatigue domain—Pediatric version and Adult version*	*	T-score metric (M = 50, SD = 10), Higher scores indicate greater fatigue	14	Median (IQR)	MCV levels < 102 fl	60.5 (50–63.5)
[8]	PROMIS CAT Fatigue domain—Pediatric version and Adult version*	*	T-score metric (M = 50, SD = 10), Higher scores indicate greater fatigue	12	Median (IQR)	MCV levels > or = 102 fl	39.5 (31–50.2)
[9]	PROMIS CAT Fatigue domain—Pediatric version and Adult version*	*	T-score metric (M = 50, SD = 10), Higher scores indicate greater fatigue	10	Median (IQR)	Negative belief	53 (44–61)
[9]	PROMIS CAT Fatigue domain—Pediatric version and Adult version*	*	T-score metric (M = 50, SD = 10), Higher scores indicate greater fatigue	21	Median (IQR)	No negative belief	49 (40–61)
[9]	PROMIS CAT Fatigue domain—Pediatric version and Adult version*	*	T-score metric (M = 50, SD = 10), Higher scores indicate greater fatigue	14	Median (IQR)	Recall barrier	59 (45–64)
[9]	PROMIS CAT Fatigue domain—Pediatric version and Adult version*	*	T-score metric (M = 50, SD = 10), Higher scores indicate greater fatigue	17	Median (IQR)	No recall barrier	46 (35–54)
[9]	PROMIS CAT Fatigue domain—Pediatric version and Adult version*	*	T-score metric (M = 50, SD = 10), Higher scores indicate greater fatigue	10	Median (IQR)	Access barrier	48 (44–61)
[9]	PROMIS CAT Fatigue domain—Pediatric version and Adult version*	*	T-score metric (M = 50, SD = 10), Higher scores indicate greater fatigue	21	Median (IQR)	No access barrier	51 (40–61)

PROMIS Adult Fatigue Measures							
Reference	Type	Nb of items	Interpretation/Cut-off/Calibration	N total	Report type	Score condition	Score
[18]	PROMIS Adult Fatigue item bank	1*	T-score metric (M = 50, SD = 10), Higher scores indicate greater fatigue	2201	Mean (SD)	Total sample	55.5 (9.4)
[22]	PROMIS Adult Fatigue Domain Full Bank	*	T-score metric (M = 50, SD = 10), Higher scores indicate greater fatigue, Thresholds: 0.5, 1.0, 2.0 SDs*	14	Median (IQR)	Chronic pain	60.7 (56.5–69.7*)
[22]	PROMIS Adult Fatigue Domain Full Bank	*	T-score metric (M = 50, SD = 10), Higher scores indicate greater fatigue, Thresholds: 0.5, 1.0, 2.0 SDs*	19	Median (IQR)	No chronic pain	52.8 (45.2–60.4)
[3]	PROMIS Fatigue SF	7	Scores can range from 7 to 35*, Higher scores indicate greater fatigue	60	Range of scores (Min–Max)	Total sample	8–30
[4]	PROMIS Fatigue SF	7	Scores can range from 7 to 35*, Higher scores indicate greater fatigue	60	Mean (SD)	PROMIS Fatigue SF total	19.82 (5.3)
[16]	PROMIS Fatigue SF	7	T-score metric (M = 50, SD = 10), Higher scores indicate greater fatigue	45	Mean (SD)	Total sample	55.93 (9.47)
[16]	PROMIS Fatigue SF	7	T-score metric (M = 50, SD = 10), Higher scores indicate greater fatigue	20	Mean (SD)	Disease Severity: Mild	55.20 (9.74)
[16]	PROMIS Fatigue SF	7	T-score metric (M = 50, SD = 10), Higher scores indicate greater fatigue	25	Mean (SD)	Disease Severity: Severe	56.51 (9.42)
[16]	PROMIS Fatigue SF	7	T-score metric (M = 50, SD = 10), Higher scores indicate greater fatigue	17	Mean (SD)	HU	52.68 (10.14)
[16]	PROMIS Fatigue SF	7	T-score metric (M = 50, SD = 10), Higher scores indicate greater fatigue	10	Mean (SD)	Chronic Transfusions	58.32 (7.36)
[16]	PROMIS Fatigue SF	7	T-score metric (M = 50, SD = 10), Higher scores indicate greater fatigue	19	Mean (SD)	No disease-modifying therapy	57.55 (9.25)
[17]	PROMIS Fatigue SF	7	T-score metric (M = 50, SD = 10), Higher scores indicate greater fatigue	*	Mean	SCD Severity: Low	54.41
[17]	PROMIS Fatigue SF	7	T-score metric (M = 50, SD = 10), Higher scores indicate greater fatigue	*	Mean	SCD Severity: Medium	55.50
[17]	PROMIS Fatigue SF	7	T-score metric (M = 50, SD = 10), Higher scores indicate greater fatigue	*	Mean	SCD Severity: High	58.24
[19]	PROMIS Fatigue SF	7	T-score metric (M = 50, SD = 10), Higher scores indicate greater fatigue	60	Mean (SE)	PROMIS-Fatigue Score	19.08 (0.68)
[19]	PROMIS Fatigue SF	7	T-score metric (M = 50, SD = 10), Higher scores indicate greater fatigue	60	Mean (SE)	PROMIS-Fatigue T-score	55.9 (1.03)
[10]	PROMIS Fatigue SF	6	T-score metric (M = 50, SD = 10), Higher scores indicate greater fatigue, Thresholds: 0.5 SD for changes in HRQL in chronic illness*	47	Mean (?)*	Entire cohort	53.7 (46.1–60)
[10]	PROMIS Fatigue SF	6	T-score metric (M = 50, SD = 10), Higher scores indicate greater fatigue, Thresholds: 0.5 SD for changes in HRQL in chronic illness*	10	Mean (?)*	Male	45.15 (33.4–47.8)
[10]	PROMIS Fatigue SF	6	T-score metric (M = 50, SD = 10), Higher scores indicate greater fatigue, Thresholds: 0.5 SD for changes in HRQL in chronic illness*	37	Mean (?)*	Female	56.3 (49.4–61.2)
[10]	PROMIS Fatigue SF	6	T-score metric (M = 50, SD = 10), Higher scores indicate greater fatigue, Thresholds: 0.5 SD for changes in HRQL in chronic illness*	33	Mean (?)*	Pain on 3 or more days/week	56.3 (47.8–61.2)
[10]	PROMIS Fatigue SF	6	T-score metric (M = 50, SD = 10), Higher scores indicate greater fatigue, Thresholds: 0.5 SD for changes in HRQL in chronic illness*	14	Mean (?)*	Pain < 3 days/week	50.15 (44.2–53.7)
[6]	PROMIS CAT Fatigue Domain—Pediatric version and Adult version*	Vary within and in-between patients*	T-score metric (M = 50, SD = 10), Higher scores indicate greater fatigue	34	Median (IQR)	Total sample	49.4 (39.9–60.9)
[6]	PROMIS CAT Fatigue domain—Pediatric version and Adult version*	Vary within and in-between patients*	T-score metric (M = 50, SD = 10), Higher scores indicate greater fatigue	*	Median (IQR)	12–17 years	46.2 (35–57.1)
[6]	PROMIS CAT Fatigue domain—Pediatric version and Adult version*	Vary within and in-between patients*	T-score metric (M = 50, SD = 10), Higher scores indicate greater fatigue	*	Median (IQR)	18–22 years	60.7 (51–62.6)
[6]	PROMIS CAT Fatigue domain—Pediatric version and Adult version*	Vary within and in-between patients*	T-score metric (M = 50, SD = 10), Higher scores indicate greater fatigue	*	Median (IQR)	Male	45.3 (39.1–54.1)
[6]	PROMIS CAT Fatigue domain—Pediatric version and Adult version*	Vary within and in-between patients*	T-score metric (M = 50, SD = 10), Higher scores indicate greater fatigue	*	Median (IQR)	Female	57.1 (48.8–62)
[6]	PROMIS CAT Fatigue domain—Pediatric version and Adult version*	Vary within and in-between patients*	T-score metric (M = 50, SD = 10), Higher scores indicate greater fatigue	*	Median (IQR)	Chronic pain	61.5 (60.9–62)
[6]	PROMIS CAT Fatigue domain—Pediatric version and Adult version*	Vary within and in-between patients*	T-score metric (M = 50, SD = 10), Higher scores indicate greater fatigue	*	Median (IQR)	No chronic pain	49 (39.9–60)
[7]	PROMIS CAT Fatigue domain—Pediatric version and Adult version*	*	T-score metric (M = 50, SD = 10), Higher scores indicate greater fatigue	14	Median (IQR)	No hospitalizations in 1 year prior	45.3 (35.2–49.7)
[7]	PROMIS CAT Fatigue domain—Pediatric version and Adult version*	*	T-score metric (M = 50, SD = 10), Higher scores indicate greater fatigue	11	Median (IQR)	1–3 hospitalizations in 1 year prior	48.8 (35–62.4)
[7]	PROMIS CAT Fatigue domain—Pediatric version and Adult version*	*	T-score metric (M = 50, SD = 10), Higher scores indicate greater fatigue	9	Median (IQR)	Hospitalizations > or = 4 in 1 year prior	61.5 (57.1–63.1)
[8]	PROMIS CAT Fatigue domain—Pediatric version and Adult version*	*	T-score metric (M = 50, SD = 10), Higher scores indicate greater fatigue	31	Median (IQR)	Total sample	49.4 (39.9–60.9)
[8]	PROMIS CAT Fatigue domain—Pediatric version and Adult version*	*	T-score metric (M = 50, SD = 10), Higher scores indicate greater fatigue	22	Median (IQR)	Low adherence to HU (according to MMAS-8)	55.5 (45.8–62.2)
[8]	PROMIS CAT Fatigue domain—Pediatric version and Adult version*	*	T-score metric (M = 50, SD = 10), Higher scores indicate greater fatigue	22	Median (IQR)	Moderate/high adherence to HU (according to MMAS-8)	39.1 (28.3–43.7)
[8]	PROMIS CAT Fatigue domain—Pediatric version and Adult version*	*	T-score metric (M = 50, SD = 10), Higher scores indicate greater fatigue	12	Median (IQR)	Low HbF % levels < 16%	52.6 (35–62.7)
[8]	PROMIS CAT Fatigue domain—Pediatric version and Adult version*	*	T-score metric (M = 50, SD = 10), Higher scores indicate greater fatigue	14	Median (IQR)	High HbF % levels > or = 16%	47.7 (39.5–58.8)
[8]	PROMIS CAT Fatigue domain—Pediatric version and Adult version*	*	T-score metric (M = 50, SD = 10), Higher scores indicate greater fatigue	14	Median (IQR)	MCV levels < 102 fl	60.5 (50–63.5)
[8]	PROMIS CAT Fatigue domain—Pediatric version and Adult version*	*	T-score metric (M = 50, SD = 10), Higher scores indicate greater fatigue	12	Median (IQR)	MCV levels > or = 102 fl	39.5 (31–50.2)
[9]	PROMIS CAT Fatigue domain—Pediatric version and Adult version*	*	T-score metric (M = 50, SD = 10), Higher scores indicate greater fatigue	10	Median (IQR)	Negative belief	53 (44–61)
[9]	PROMIS CAT Fatigue domain—Pediatric version and Adult version*	*	T-score metric (M = 50, SD = 10), Higher scores indicate greater fatigue	21	Median (IQR)	No negative belief	49 (40–61)
[9]	PROMIS CAT Fatigue domain—Pediatric version and Adult version*	*	T-score metric (M = 50, SD = 10), Higher scores indicate greater fatigue	14	Median (IQR)	Recall barrier	59 (45–64)
[9]	PROMIS CAT Fatigue domain—Pediatric version and Adult version*	*	T-score metric (M = 50, SD = 10), Higher scores indicate greater fatigue	17	Median (IQR)	No recall barrier	46 (35–54)
[9]	PROMIS CAT Fatigue domain—Pediatric version and Adult version*	*	T-score metric (M = 50, SD = 10), Higher scores indicate greater fatigue	10	Median (IQR)	Access barrier	48 (44–61)
[9]	PROMIS CAT Fatigue domain—Pediatric version and Adult version*	*	T-score metric (M = 50, SD = 10), Higher scores indicate greater fatigue	21	Median (IQR)	No access barrier	51 (40–61)

Brief Fatigue Inventory (BFI)							
Reference	Type	Nb of items	Interpretation/Cut-off/Calibration	N total	Report type	Score condition	Score
[3]	BFI	9	Potential range (min–max): 0–10, Higher scores indicating greater fatigue, cut-off not specified*	*	*	*	*
[4]	BFI	10*	Potential range (min–max): 0–10, Higher scores indicating greater fatigue, cut-off not specified*	60	Mean (SD)	BFI Total	4.30 (2.16)
[4]	BFI	10*	Potential range (min–max): 0–10, Higher scores indicating greater fatigue, cut-off not specified*	60	Mean (SD)	Fatigue severity: Now	3.83 (2.52)
[4]	BFI	10*	Potential range (min–max): 0–10, Higher scores indicating greater fatigue, cut-off not specified*	60	Mean (SD)	Usual fatigue during the past 24 h	4.10 (2.63)
[4]	BFI	10*	Potential range (min–max): 0–10, Higher scores indicating greater fatigue, cut-off not specified*	60	Mean (SD)	Worst fatigue during the past 24 h	5.32 (2.97)
[4]	BFI	10*	Potential range (min–max): 0–10, Higher scores indicating greater fatigue, cut-off not specified*	60	Mean (SD)	Interference of fatigue	3.96 (2.38)
[21]	The Fatigue Severity Subscale of the Brief Fatigue Inventory (BFI)*	3	Potential range (min–max): 0–10, Higher scores indicating greater fatigue, cut-off not specified*	60	Mean (SD)	Fatigue severity	5.32 (2.74)

Fatigue Severity Scale (FSS)				Reported scores			
Reference	Type	Nb of items	Interpretation/Cut-off/Calibration	N total	Report type	Score condition	Score
[1]	Fatigue Severity Scale (FSS)	9	Cut-off = 36, "The higher and lower scores from 36 indicated the patients with and without signs of fatigue, respectively"	97	Mean (SD)	Fatigue severity	38.56 (14.88)
[1]	Fatigue Severity Scale (FSS)	9	Cut-off = 36, "The higher and lower scores from 36 indicated the patients with and without signs of fatigue, respectively"	34	Number (%)	No signs of fatigue (score < 36)	34 (35.1)
[1]	Fatigue Severity Scale (FSS)	9	Cut-off = 36, "The higher and lower scores from 36 indicated the patients with and without signs of fatigue, respectively"	63	Number (%)	Signs of fatigue (score > 36)	63 (64.9)
[2] *	Fatigue Severity Scale (FSS)	9	Cut-off = 36, "Those with a score of 36 and higher were considered to have symptoms of fatigue; those with scores less than 36 were considered to have no signs of fatigue"	53	Mean (SD)	Baseline Final Sample	36.47 (13.33)
[2] *	Fatigue Severity Scale (FSS)	9	Cut-off = 36, "Those with a score of 36 and higher were considered to have symptoms of fatigue; those with scores less than 36 were considered to have no signs of fatigue"	29	Mean (SD)	Dropouts	44.86 (11.79)
[2]	Fatigue Severity Scale (FSS)	9	Cut-off = 36, "Those with a score of 36 and higher were considered to have symptoms of fatigue; those with scores less than 36 were considered to have no signs of fatigue"	53	Mean (SD)	36 weeks later (12 weeks of intervention + 24 weeks of follow-up)	18.5 (13.19)
[2]	Fatigue Severity Scale (FSS)	9	Cut-off = 36, "Those with a score of 36 and higher were considered to have symptoms of fatigue; those with scores less than 36 were considered to have no signs of fatigue"	23	Number (%)	No signs of fatigue (score < 36)	23 (43.4)
[2]	Fatigue Severity Scale (FSS)	9	Cut-off = 36, "Those with a score of 36 and higher were considered to have symptoms of fatigue; those with scores less than 36 were considered to have no signs of fatigue"	30	Number (%)	Signs of fatigue (score > 36)	30 (56.6)

Multi-dimensional Fatigue Inventory (MFI)				Reported scores			
Reference	Type	Nb of items	Interpretation/Cut-off/Calibration	N total	Report type	Score condition	Score
[28]	Multi-dimensional Fatigue Inventory (MFI)	*	Value range: 0–100,	13	Mean (SD)	Syndrome 1 diagnosed in Traditional Chinese Medicine (TCM #1)	45.58 (9.395)
[28]	Multi-dimensional Fatigue Inventory (MFI)	*	Value range: 0–100, no cut-off specified*	20	Mean (SD)	Syndrome 2 diagnosed in Traditional Chinese Medicine (TCM #2)	56.00 (19.73)
[28]	Multi-dimensional Fatigue Inventory (MFI)	*	Value range: 0–100, no cut-off specified*	19	Mean (SD)	Syndrome 3 diagnosed in Traditional Chinese Medicine (TCM #3)	61.60 (15.29)

Multidimensional Fatigue Symptom Inventory-Short Form (MFSI-SF)				Reported scores			
*Reference*	*Type*	*Nb of items*	*Interpretation/Cut-off/Calibration*	*N total*	*Report type*	*Score condition*	*Score*
[3]	Multidimensional Fatigue Symptom Inventory-Short Form (MFSI-SF)	30	Potential range (min–max): 24–86*, Higher scores indicating greater fatigue	*	*	*	*
[4]	Multidimensional Fatigue Symptom Inventory-Short Form (MFSI-SF)	30	Potential range (min–max): − 24 to 86*	60	Mean (SD)	MFSI-SF total	14.89 (20.3)
[4]	Multidimensional Fatigue Symptom Inventory-Short Form (MFSI-SF)	6	Potential range (min–max): − 24 to 86*	60	Mean (SD)	General Fatigue	9.13 (5.7)
[4]	Multidimensional Fatigue Symptom Inventory-Short Form (MFSI-SF)	6	Potential range (min–max): − 24 to 86*	60	Mean (SD)	Emotional Fatigue	6.83 (5.6)
[4]	Multidimensional Fatigue Symptom Inventory-Short Form (MFSI-SF)	6	Potential range (min–max): − 24 to 86*	60	Mean (SD)	Physical Fatigue	4.75 (4.8)
[4]	Multidimensional Fatigue Symptom Inventory-Short Form (MFSI-SF)	6	Potential range (min–max): − 24 to 86*	60	Mean (SD)	Mental Fatigue	5.52 (4.9)
[4]	Multidimensional Fatigue Symptom Inventory-Short Form (MFSI-SF)	6	Potential range (min–max): − 24 to 86*	60	Mean (SD)	Vigor	11.35 (5.4)

Pediatric quality of life multidimensional fatigue scale (PedsQL™ MFS)				Reported scores			
Reference	Type	Nb of items	Interpretation/cut-off/calibration	N total	Report type	Score condition	Score
[5]	PedsQL™ MFS	18	Potential range (min–max): 0–100, Lower scores indicating more fatigue	32	Mean (SD)	Summary Fatigue (child report)	67.5 (20.0)
[5]	PedsQL™ MFS	6	Potential range (min–max): 0–100, Lower scores indicating more fatigue	32	Mean (SD)	General Fatigue (child report)	72.7 (21.3)
[5]	PedsQL™ MFS	6	Potential range (min–max): 0–100, Lower scores indicating more fatigue	32	Mean (SD)	Sleep/Rest Fatigue (child report)	61.43 (23.3)
[5]	PedsQL™ MFS	6	Potential range (min–max): 0–100, Lower scores indicating more fatigue	32	Mean (SD)	Cognitive Fatigue (child report)	68.0 (26.8)
[13]	PedsQL™ MFS	18	Potential range (min–max): 0–100, Lower scores indicating more fatigue	1393	Mean (SD)	Total Fatigue (child report)	69.04 (18.11)
[13]	PedsQL™ MFS	6	Potential range (min–max): 0–100, Lower scores indicating more fatigue	1392	Mean (SD)	General Fatigue (child report)	72.96 (20.24)
[13]	PedsQL™ MFS	6	Potential range (min–max): 0–100, Lower scores indicating more fatigue	1391	Mean (SD)	Sleep/Rest Fatigue (child report)	64.87 (21.60)
[13]	PedsQL™ MFS	6	Potential range (min–max): 0–100, Lower scores indicating more fatigue	1390	Mean (SD)	Cognitive Fatigue (child report)	69.33 (23.97)
[23]	PedsQL™ MFS	18	Potential range (min–max): 0–100, Lower scores indicating more fatigue	157	Mean (SD)	ED Visit—Total Fatigue (child report)	53.8 (18.53)
[23]	PedsQL™ MFS	6	Potential range (min–max): 0–100, Lower scores indicating more fatigue	158	Mean (SD)	ED Visit—General Fatigue (child report)	52.0 (22.20)
[23]	PedsQL™ MFS	6	Potential range (min–max): 0–100, Lower scores indicating more fatigue	157	Mean (SD)	ED Visit—Sleep/Rest Fatigue (child report)	48.6 (20.65)
[23]	PedsQL™ MFS	6	Potential range (min–max): 0–100, Lower scores indicating more fatigue	155	Mean (SD)	ED Visit—Cognitive Fatigue (child report)	60.3 (26.29)
[23]	PedsQL™ MFS	18	Potential range (min–max): 0–100, Lower scores indicating more fatigue	134	Mean	1 week—Total Fatigue (child report)	61.0
[23]	PedsQL™ MFS	6	Potential range (min–max): 0–100, Lower scores indicating more fatigue	135	Mean	1 week—General Fatigue (child report)	60.0
[23]	PedsQL™ MFS	6	Potential range (min–max): 0–100, Lower scores indicating more fatigue	134	Mean	1 week—Sleep/Rest Fatigue (child report)	57.1
[23]	PedsQL™ MFS	6	Potential range (min–max): 0–100, Lower scores indicating more fatigue	132	Mean	1 week—Cognitive Fatigue (child report)	65.2
[23]	PedsQL™ MFS	18	Potential range (min–max): 0–100, Lower scores indicating more fatigue	144	Mean	1–3 months—Total Fatigue (child report)	62.1
[23]	PedsQL™ MFS	6	Potential range (min–max): 0–100, Lower scores indicating more fatigue	145	Mean	1–3 months—General Fatigue (child report)	64.0
[23]	PedsQL™ MFS	6	Potential range (min–max): 0–100, Lower scores indicating more fatigue	144	Mean	1–3 months—Sleep/Rest Fatigue (child report)	59.2
[23]	PedsQL™ MFS	6	Potential range (min–max): 0–100, Lower scores indicating more fatigue	142	Mean	1–3 months—Cognitive Fatigue (child report)	63.2
[24]	PedsQL™ MFS	18	Higher scores indicating better HRQOL (lower fatigue symptoms)	240	Mean (SD)	Total Fatigue (child report)	61.1 (19.8)
[24]	PedsQL™ MFS	6	Higher scores indicating better HRQOL (lower fatigue symptoms)	240	Mean (SD)	General Fatigue (child report)	65.4 (22.7)
[24]	PedsQL™ MFS	6	Higher scores indicating better HRQOL (lower fatigue symptoms)	240	Mean (SD)	Sleep/Rest Fatigue (child report)	58.2 (23.3)
[24]	PedsQL™ MFS	6	Higher scores indicating better HRQOL (lower fatigue symptoms)	240	Mean (SD)	Cognitive Fatigue (child report)	59.8 (24.4)
[24]	PedsQL™ MFS	18	Higher scores indicating better HRQOL (lower fatigue symptoms)	104	Mean (SD)	Severe SCD—Total Fatigue (child report)	59.8 (21.4)
[24]	PedsQL™ MFS	6	Higher scores indicating better HRQOL (lower fatigue symptoms)	104	Mean (SD)	Severe SCD—General Fatigue (child report)	63.4 (24.9)
[24]	PedsQL™ MFS	6	Higher scores indicating better HRQOL (lower fatigue symptoms)	104	Mean (SD)	Severe SCD—Sleep/Rest Fatigue (child report)	58.3 (22.3)
[24]	PedsQL™ MFS	6	Higher scores indicating better HRQOL (lower fatigue symptoms)	104	Mean (SD)	Severe SCD—Cognitive Fatigue (child report)	57.9 (25.4)
[24]	PedsQL™ MFS	18	Higher scores indicating better HRQOL (lower fatigue symptoms)	136	Mean (SD)	Mild SCD—Total Fatigue (child report)	62.1 (18.5)
[24]	PedsQL™ MFS	6	Higher scores indicating better HRQOL (lower fatigue symptoms)	136	Mean (SD)	Mild SCD—General Fatigue (child report)	66.9 (21.0)
[24]	PedsQL™ MFS	6	Higher scores indicating better HRQOL (lower fatigue symptoms)	136	Mean (SD)	Mild SCD—Sleep/Rest Fatigue (child report)	58.1 (24.1)
[24]	PedsQL™ MFS	6	Higher scores indicating better HRQOL (lower fatigue symptoms)	136	Mean (SD)	Mild SCD—Cognitive Fatigue (child report)	61.3 (23.6)
[25]	PedsQL™ MFS	6	Potential range (min–max): 0–100, Lower scores indicating more fatigue	18	Mean (SD)	General Fatigue (participant report)	61.0 (19.0)
[25]	PedsQL™ MFS	6	Potential range (min–max): 0–100, Lower scores indicating more fatigue	18	Mean (SD)	Sleep/Rest Fatigue (participant report)	52.2 (22.7)
[25]	PedsQL™ MFS	6	Potential range (min–max): 0–100, Lower scores indicating more fatigue	18	Mean (SD)	Cognitive Fatigue (participant report)	52.9 (22.4)
[26]	PedsQL™ MFS	*	Potential range (min–max): 0–100, Higher scores indicating lower fatigue	106	Mean (SD)	PedsQL Fatigue (self-report)	64 (18.1)
[27]	PedsQL™ MFS	*	Potential range (min–max): 0–100, Lower scores indicating more fatigue	38	Mean (SD) for raw score*	Total Fatigue (child self-report)	72 (9)
[27]	PedsQL™ MFS	*	Potential range (min–max): 0–100, Lower scores indicating more fatigue	38	Mean (SD) for raw score*	General Fatigue (child self-report)	76 (15)
[27]	PedsQL™ MFS	*	Potential range (min–max): 0–100, Lower scores indicating more fatigue	38	Mean (SD) for raw score*	Cognitive Fatigue (child self-report)	70 (15)

*See appendix for methodological issues

Most studies employing the PROMIS tool adopted various versions (full bank, short forms, CAT versions) that generate a single global fatigue score interpreted according to a T-score metric (with a mean of 50 and a standard deviation of 10), with a higher score indicates greater fatigue. This approach limits the ability to differentiate the experience and impact of fatigue across different dimensions. Consequently, data on the influence of fatigue on specific aspects of daily functioning is scarce. Furthermore, aggregating fatigue scores from the PROMIS system into a meaningful average is difficult. Studies not only use different PROMIS versions but also employed various statistical measures for reporting scores (e.g., T-scores, raw scores, means, medians, ranges). Moreover, certain studies used a combination of different versions, selected only a few items from a single version or did not specify the version or number of items used, making the employed instrument unique or impossible to identify and consequently challenging to compare with other results.

Other methodological issues concerning the PROMIS tool and other instruments (see appendix for further details), also contribute to the difficulty in concluding on the prevalence of fatigue in this population, such as a lack of information on the number of participants per condition analysed, or the absence of information on the method of interpretation or calibration of the results, or when a threshold or cut-off score was not indicated.

The Pediatric Quality of Life Inventory Multidimensional Fatigue Scale (PedsQL™ MFS) represents a partial exception to the general trend. It offers a total fatigue score (except for one study) with a wider range reported across studies (53.8–72). Furthermore, the data on individual dimension scores demonstrate a degree of consistency, especially for general and cognitive fatigue, which exhibit methodologically comparable scores in six out of seven studies.

However, the remaining instruments, each with unique fatigue assessment approaches, are less well represented in the review, making comparisons of their scores even more challenging. It’s important to note that a consistent feature across most of the instruments is that higher scores signify greater fatigue severity, except for the PedsQL™ MFS where higher scores indicate less fatigue.

### Correlates of fatigue

General consideration: Beyond the assessment of fatigue, 26 studies explored various potential associations with other health outcomes. Pain or pain-related variables (n = 12), and medical or biological outcomes (n = 13) were the most frequently examined correlates. Less frequent, but still explored in some studies, correlates include hospitalisations and emergency department visits (n = 4), sociodemographic characteristics (n = 8), psychological and social outcomes (n = 8), quality of life or health-related quality of life (QOL or HRQOL) domains (n = 6), disease-related outcomes (n = 9), neurocognitive outcomes (n = 5) and miscellaneous outcomes such as school/work absences, adherence and barriers to hydroxyurea/hydroxycarbamide (n = 3).

Medical or biological outcomes: it is noteworthy that bone marrow transplants were not explicitly investigated as a correlate of fatigue in any of the studies reviewed. Only three studies made brief mention of transplants: Panepinto et al. [33] referenced them in establishing disease severity criteria; Bakshi et al. [40] included them among other treatment considerations; Knisely et al. [41] excluded patients who had undergone successful bone marrow transplant. However, none of these studies explored the potential associations between transplant history and fatigue. For the remaining studies, the allogenic stem cell transplantation status of the patient cohorts remains unclear, although it remains the only curative treatment so far [42].

Associations examined based on fatigue assessment role: The studies categorised fatigue as a primary, secondary, or instrument-development outcome, and the investigated associations differed accordingly. When fatigue was the main focus, studies primarily examined its association with psychological and social outcomes (n = 5), along with medical/biological outcomes (n = 5), and disease-related outcomes (n = 4).

As a secondary outcome, fatigue was most often investigated in relation to pain-related dimension (n = 5), medical/biological outcomes (n = 5) and sociodemographic outcomes (n = 4).

Study focused on developing, testing, validating or comparing fatigue assessment tools primarily evaluate fatigue with pain or pain-related variables (n = 4), medical or biological outcomes (n = 3) and disease-related outcomes (n = 4).

Inconsistencies in associations: It is important to note that the studies yielded mixed findings regarding the significance of these associations. For example, the relationship between fatigue and pain, disease severity, or sociodemographic variables (like age and gender) showed inconsistencies across studies, with some reporting significant associations and others not.

## Discussion

Given the lack of a standardised definition and measurement approach for fatigue in sickle cell disease (SCD) within the existing literature, clinicians and researchers face significant challenges in selecting appropriate assessment tools. As the study of fatigue in SCD is a relatively new field, it is imperative to acquire a comprehensive understanding of how this symptom has been evaluated in recent investigations. Consequently, the primary objective of this systematic literature review was to provide a systematic overview of the currently employed self-reported psychometric measures, their dimensions, and the levels of fatigue assessed in SCD. Additionally, the review sought to identify the associated variables investigated in the selected studies. The thorough examination of 28 articles led to the successful cataloguing of 16 instruments used with children, adolescents, and adults afflicted with SCD. Overall, the review demonstrates the lack of standardisation in the assessment of fatigue, both in terms of the instruments used and the dimensions measured. Consequently, no synthetised data on fatigue (prevalence or intensity) could be extracted.

While fatigue has been widely recognised as a significant symptom in SCD [9–13], a notable absence of instruments specifically tailored to measure fatigue in this context has persisted over the past fourteen years. This observation lends credence to the notion that fatigue remains an understudied symptom in the SCD literature [10], potentially indicating a limited understanding and precise definition within this pathology. Among the studies included in our review, fatigue was infrequently examined as an isolated symptom or considered as a primary outcome, often being included into broader assessment of multiple symptoms. This finding suggested a potential lack of dedicated research interest in fatigue relative to other prominent symptoms, such as pain, leading to its underrepresentation in the scientific literature.

In the absence of a dedicated tool, researchers investigating fatigue in SCD patients have employed a diverse array of instruments, including the PROMIS system (represented by ten distinct versions), the BFI, the FSS, the MFI-20, the MFSI-SF, and the PedsQL™ MFS. Few of these identified instruments have been validated in the target population (e.g. MFSI-SF, PedsQL™ MFS, BFI, PROMIS SF and paediatric forms) or in groups of patients with chronic pathologies. As there is no consensus within the scientific community regarding the most appropriate approach for assessing fatigue in SCD, the frequency of use for these instruments varies significantly. Among the analysed studies, the PROMIS system emerged as a particularly prominent tool. This prevalence can be attributed to several distinctive qualities and advantages, including its rigorous development methodology [28, 43], extensive literature supporting its reliability and validity, and its Computerised Adaptive Testing (CAT) method, which enable adaptative questioning to the patient's responses to enhance efficiency and accuracy [44]. Another notable advantage of the PROMIS system is its extensive item bank, allowing for the creation of tailored versions to accommodate diverse populations and assessment contexts. Moreover, the system’s capability to develop instruments specifically adapted to particular pathologies, as demonstrated by tools for conditions such as multiple sclerosis [45] and fibromyalgia [46], suggests the potential benefits of developing a dedicated PROMIS version for SCD. However, a limitation of the PROMIS system lies in its unidimensionality and the generation of a single fatigue score. Given that the PROMIS system measures overall fatigue, it may not fully capture the multifaceted nature of fatigue experiences and their various impacts.

Beyond the PROMIS versions, our review identified other unidimensional tools, as well as a few multidimensional instruments. While unidimensional tools predominate in our sample of studies, the presence of both types suggests a lack of consensus regarding the dimensional nature of fatigue in SCD. This finding further emphasises that the choice of a measurement tool depends not only on the pathology under investigation but also on the specific research objectives and preference of the researchers [17]. As noted by several authors, different measures may be appropriate for evaluating fatigue severity, screening for fatigue, or comprehensively assessing its experience and impact on patients. While unidimensional measures may suffice for screening or assessing severity, multidimensional tools are more suitable for a comprehensive evaluation. Researchers often balance the need for precise measurements with practical consideration such as ease and duration of administration as a function of scale length [22, 23]. When comparing the assessment of fatigue to the evaluation of another hallmark symptom of SCD, pain, we observe that both intensity and functional impact are typically evaluated [47]. A similar approach may be warranted for fatigue. Our review demonstrates that this approach has already been adopted by researchers utilising multidimensional tools like the MFI-20, the MFSI-SF and the PedsQL™ MFS. These instruments assess both the severity of fatigue and its impact across various dimensions, generating distinct fatigue scores that provide a holistic understanding of the symptom in the context of SCD. In one particular study, researchers combined a multidimensional tool like the MFSI-SF with two additional unidimensional tools (the BFI and the PROMIS Fatigue SF 7-item) to measure the overall fatigue experience [11]. While this methodology can be effective, it may be time-consuming for the participants. The underrepresentation of multidimensional tools in our sample may be partially attributed to their increased administration time due to their larger number of items. Additionally, the rationale for evaluating different aspects of fatigue in SCD may not have been sufficiently established and could be further developed if research contributed to a precise definition of fatigue in this disease. Pending further investigations, we propose that employing a combination of unidimensional and multidimensional tools represents a valuable compromise in the effort to best capture the fatigue experience of patients with SCD. To this end, researchers and clinicians might consider using a unidimensional instrument such as the FSS [37] to assess fatigue severity as a primary criterion—benefiting from the availability of a cut-of score—while complementing this with the MFI or MFI-20 [39] to evaluate the broader impact of fatigue across multiple dimensions. Our recommendation of the latter tool is particularly informed by its validation in French [48].

Additionally, the literature recommends that researchers and clinicians carefully consider the specific aspects or dimensions of fatigue that require evaluation and articulate the rational for their selection [19]. While the three multidimensional tools identified in this review assess various dimensions of fatigue, their authors often provided insufficient clarity regarding these dimensions’ definition, relying on examples to illustrate them. This lack of clarity can hinder the interpretation of results across different dimensions. For instance, the distinctions between mental fatigue and cognitive fatigue, assessed by the MFI and the MFSI-SF for mental fatigue and assessed by the PedsQL™ MFS for cognitive fatigue, is not well-defined. Both dimensions refer to cognitive symptoms and concentration difficulties, making it challenging to differentiate them. According to Billones et al. [17], distinguishing between “mentally strenuous tasks” and “cognitive activities” is challenging, despite cognitive fatigue being characterised by its overwhelming and persistent nature. Their scoping review highlights the interchangeable use of these dimensions, indicating potential confusion. Studies have also considered mental fatigue as encompassing emotional and motivational aspects of fatigue, while others view cognitive fatigue as a component of mental fatigue. In the context of assessing fatigue in SCD, Panepinto et al. [33] have previously emphasised the need for further research to clarify whether cognitive fatigue, as assessed by the PedsQL™ MFS, primarily relates to attentional or cognitive difficulties resulting from a stroke or to mental fatigue. Ultimately, the selection of a fatigue assessment tool requires a precise understanding of the fatigue phenomenon and its underlying causes within the specific pathology [19]. While some factors contributing to fatigue in SCD have been identified [9, 11], further exploration of these factors is warranted.

One of the well-established factors contributing to fatigue in SCD is anaemia [6]. In this specific context with anaemic patients, researchers have highlighted a critical limitation of fatigue as an outcome measure, which warrants attention here [49]. As a self-reported subjective measure, the assessment of fatigue, as conducted by the various tools presented, does not account for the patients' activity level and how their activity may influence their level of fatigue. Consequently, this measure fails to distinguish between patients with similar fatigue levels who may differ in terms of functional capacities and activity levels. To address these factors, a measure of fatigability, that is, a measure of self-reported fatigue in the context of a standardised activity level, would therefore provide a more accurate means of capturing the patient's experience of fatigue while accounting for the relationship between fatigue and activity. Thus, measuring fatigability in patients could allow for a more precise identification of those whose fatigue significantly interferes with their normal activities. Fatigability scales that have already been used with anaemic patients include the Pittsburgh Fatigability Scale (PFS) and the Situational Fatigue Scale (SFS). These could be valuable tools to combine with fatigue assessment tools in SCD context.

The comparison and interpretation of fatigue scores reported in the studies were hindered by several factors. The use of various instruments to investigate fatigue in SCD introduced significant variability in the results, making direct comparisons and potential meta-analyses challenging. From a methodological perspective, the studies employed different statistical approaches to report scores, and in some cases, the specific methodology was not disclosed [29, 40]. Additionally, the heterogeneity of participants recruited from various healthcare settings contributed to the difficulty in directly comparing scores. We posit that the environment can influence fatigue assessment. Hospitalised participants are unlikely to exhibit fatigue levels comparable to asymptomatic participants recruited during routine visits. Hospitalisation or emergency department visits were also investigated as correlates of fatigue in some of the identified studies. These variables warrant careful consideration and further investigation when evaluating fatigue in SCD.

Among the other identified associations, our findings highlight the prominence of pain and its related variables in the studies, alongside medical or biological outcomes. This can be attributed to the well-documented nature of pain as a hallmark symptom of SCD symptom and its physical manifestations, which often lead to a cascade of other symptoms and conditions requiring medical intervention. Furthermore, the inconsistencies observed among these associations may be partially explained by the lack of standardisation in the assessment of fatigue. These inconsistencies underscore the need for further research to achieve more robust results concerning the variables associated with fatigue and to consider during its assessment.

To our knowledge, this systemic review represents the first comprehensive examination of fatigue assessment instruments, and their dimensions specifically used in SCD. It provides a unique overview of the different dimensions of fatigue assessed by these tools and offers an insight into the instruments utilised with adult and paediatric patients over the past 14 years. A notable strength of this study lies in the substantial number of systematically reviewed studies, ensuring a comprehensive coverage of the relevant research published in peer-reviewed journals. However, certain limitations exist. While searches were carried out across several databases, this review did not cover grey literature and was unable to include inaccessible studies at the article screening stage in April 2024 [50–52]. Nevertheless, to maintain the reliability of the included results, we restricted our analysis to peer-reviewed journal articles. Additionally, this review focused solely on self-reported measures of fatigue. Given the subjective and objective aspects of fatigue, future researchers should examine objective measures and peer-reported measures in this context. Furthermore, our analysis was confined to English and French language studies, potentially limiting the generalisability of our interpretation. Moreover, the predominance of American articles in our sample primarily provides American data on fatigue assessment. Considering the unique characteristics of the US healthcare system, the generalisation of these results to the French healthcare system may be limited. Given that only one European study was included, it is imperative to encourage further research on fatigue in SCD within the French and European contexts. Also, given the often underprivileged status of population affected by SCD worldwide, it is essential to avoid underrepresentation in the scientific literature. Open access should be prioritized for studies focusing on this disease and its symptoms.

A further limitation of this review is its cataloguing the diverse tools employed without providing a comprehensive analysis of their psychometrics properties. Although Cronbach’s alpha is presented for internal consistency, its use remains a subject of methodological debated [53]. Nonetheless, this work can serve as a foundation for future research to initiate a critical reflection on the selection of suitable tools for measuring fatigue in SCD populations. Moreover, this review underscores the need for the development and validation of SCD-specific measures of fatigue. To advance this field, future research should endeavour to elucidate the precise nature of fatigue in SCD, and to identify the most effective methods for its measurement.

## Conclusion

Assessing fatigue in individuals with sickle cell disease (SCD) presents a challenge due to its subjective, objective, and multifaceted nature. In addition to the fact that a precise and tailored assessment is essential for effective management of this symptom, providing patients with a detailed analysis of their fatigue could also encourage a process of empowerment by enhancing their literacy skills. Psychometric measures of fatigue can be employed with patients of varying age groups. While these measures offer a reasonable assessment of this symptom, a clearer understanding of the intensity and impact of fatigue in SCD is hindered by a dearth of relevant data. Given the pivotal role of fatigue in this pathology, the development of a specifically tailored instrument is imperative. In the absence of such a tool, future research should contemplate the utilisation of a combination of existing measures to comprehensively and representatively capture the patients' experiences.

## Supplementary Information

Additional file 1.

## Data Availability

The data that support the findings of this study are not publicly available. They are, however, available upon restrictions from the corresponding author upon reasonable request. The reuse of data is subject to compliance with the GDPR and French regulations.

## Abbreviations

AYA: Adolescents and young adults
BFI: Brief Fatigue Inventory
CAT: Computerised Adaptive Testing
FSS: Fatigue Severity Scale
HRQOL (or QOL): Health-related quality of life (or quality of life)
HU: Hydroxyurea/hydroxycarbamide use
IQR: Interquartile range
MFI (or MFI-20): Multidimensional Fatigue Inventory
MFSI-SF: Multidimensional Fatigue Symptom Inventory – Short Form
NIH: National Institutes of Health
PEDSQL™ MFS: Pediatric Quality of Life Multidimensional Fatigue Scale
PFS: Pittsburgh Fatigability Scale
PRISMA: Preferred Reporting Items for Systematic Reviews and Meta-Analyses
PROMIS: Patient-Reported Outcomes Measurement Information System
SCD: Sickle cell disease
SD: Standard deviation
SE: Standard error
SF: Short Form
SFS: Situational Fatigue Scale
