# Supplementary material for: Measurement of fatigue in sickle cell disease: a systematic review of fatigue measures
Source: Orphanet J Rare Dis. 2025 Sep 3;20:475. doi: 10.1186/s13023-025-03961-4 (PMC12409949; doi:10.1186/s13023-025-03961-4)
Supplement: Supplementary file 1 — Additional file 1. [file 13023_2025_3961_MOESM1_ESM.docx]

# Measurement of Fatigue in Sickle Cell Disease: A Systematic Review of Fatigue Measures – Supplementary material

# Methodological issues relating to the reporting of fatigue scores

PROMIS Pediatric Fatigue Measures:

- Use of a combination of full bank and short form (SF) versions without being able to distinguish between the two in the scores reported. In the studies concerned ([11], [14]), some participants responded to the full bank version and others to the SF version,
- No information on the version of the tool used (short form or full bank) or the number of items ([12]). Some studies specify the version of the tool used but do not indicate the number of items ([11], [15]),
- Lack of information on the number of participants for each condition ([11], [6]),
- Use of different methods to report scores, depending on the study: Mean (SD), Mean (SE), Range of scores (Min-Max) or Median (IQR),
- Lack of explanation of the calibration method and the parameters used to analyse the results ([20]),
- Lack of precision regarding the origin of the different scores obtained with the type of CAT measure: two studies ([6], [7]) did not specify whether the measure used came from the paediatric or adult CAT version. However, two other studies involving the same lead author a year earlier ([8], [9]) and the same sample of participants, specify as a limitation of their study that both types of CAT measures (paediatric and adult) were analysed simultaneously,
- Lack of item information for the CAT version (range of items (min-max) and mean) in four studies ([6], [7], [8], [9]),
- Several versions and number of items are possible under the name ‘short form’ (SF), for example the use of 4 items for the assessment of fatigue contained in the PROMIS Pediatric Profile 25 items - Fatigue is considered as SF ([20]), as well as the PROMIS SF version used in another study ([14]) which contains 10 items,
- Two studies conducted by the same lead author ([6] and [8]) reported the same fatigue scores for their total sample but did not mention the same number of participants (34 versus 31, respectively).

PROMIS Adult Fatigue Measures:

- Fatigue score obtained with a single item from the PROMIS Adult Fatigue item bank ([18]),
- Thresholds cited and used in two study ([22], [10]) that do not appear explicitly in other studies using PROMIS Measures,
- Use of different methods to report scores, depending on the study: Mean (SD), Median (IQR), Range of scores (Min-Max), Mean (without SD) or Mean (SE)
- The interquartile range (IQR) of a reported fatigue score is not identical in the article ([22]) and in the supplementary material of this study,
- Lack of information on the number of items contained in the tool mentioned ([22]),
- Reporting and analysis of raw fatigue scores for the 7 items and not as a function of the T score metric ([3], [4]),
- Lack of information on the number of participants for each condition ([17]),
- Lack of information on the specific methodology chosen to report scores (except for the mean) ([10])

Brief Fatigue Inventory (BFI):

- Cut-offs/thresholds not specified ([3], [4], [21]) even though they were cited by the tool's author (Mendoza et al., 1999): for the BFI, scores are categorized as ‘Mild’ (1–3), ‘Moderate’ (4–6), and ‘Severe’ (7–10),
- No scores reported for SCD in the study [3], even in the supplements to the article (but scores can be found in the article by the same principal author ([4]) on the same sample of participants).
- Error in the number of items announced (9 items instead of 10 announced because the authors counted the first question ‘have you felt unusually tired or fatigue in the last week?’ as the first item, and error in the rating scale (11 points in studies [3] and [4] instead of 10 according to the author of the scale),
- Use of a single subscale of the BFI (only severity of fatigue assessed, not interference), so only 3 items and not 9 items for study [21].

Fatigue Severity Scale (FSS):

- Use of different methods to report scores: Mean (SD) and Number (%) by threshold score
- Error in the rating scale cited (‘0 for lack of fatigue to 9 for severe fatigue’ in study [2] instead 7-point Likert scale ranging from 1 to 7 according to the author of the scale).

Multi-dimensional Fatigue Inventory (MFI):

- Lack of information on the number of items contained in the tool mentioned ([28]),
- No scores for the different subscales (dimensions of fatigue) assessed by the tool ([28]), only a total mean score and standard deviation,
- Interpretation of fatigue scores with this tool not specified ([28]), but according to the tool's author, higher scores indicate greater fatigue (without associated threshold scores).

Multidimensional Fatigue Symptom Inventory-Short Form (MFSI-SF):

- No scores reported for SCD in the study [3], even in the supplements to the article (but scores can be found in the article by the same principal author ([4]) on the same sample of participants).
- Error on the potential range of fatigue scores with this tool in studies concerned ([3],[4]): the potential range of fatigue scores is between -24 and 96 (and no -24-86 or 24-86).

Pediatric Quality of Life Multidimensional Fatigue Scale (PedsQL™ MFS):

- Use of different methods to report scores: Mean (SD), Mean only or Mean (SD) for raw score,
- Lack of information on the number of items contained in the tool mentioned ([26], [27]),
- Partially reported scores: sometimes only scores by dimension without total fatigue score ([25]), or total score reported without scores for different dimensions ([26]). Also, in one specific study [27], the authors chose not to present the scores for one dimension (Sleep/Rest Fatigue) because Cronbach's alpha was not good.

# References included in the review (alphabetical order)

1. Ahmadi, M., Poormansouri, S., Beiranvand, S., & Sedighie, L. (2018). Predictors and Correlates of Fatigue in Sickle Cell Disease Patients. *International Journal of Hematology-Oncology and Stem Cell Research, 12*(1), 69–76.
2. Ahmadi, M., Shariati, A., Poormansouri, S., & Hazeghi, N. (2015). The Effectiveness of Self Management Program on Pain, Fatigue, Depression, Anxiety, and Stress in Sickle Cell Patients: A Quasi-Experimental Study. *Jundishapur Journal of Chronic Disease Care, 4*(4). https://doi.org/10.17795/jjcdc-29521
3. Ameringer, S., Elswick, R. K., Menzies, V., Robins, J. L., Starkweather, A., Walter, J., Gentry, A. E., & Jallo, N. (2016). Psychometric Evaluation of the Patient-Reported Outcomes Measurement Information System Fatigue-Short Form Across Diverse Populations. *Nursing Research, 65*(4), 279–289. https://doi.org/10.1097/NNR.0000000000000162
4. Ameringer, S., R. K. Elswick, J. R., & Smith, W. (2014). Fatigue in Adolescents and Young Adults with Sickle Cell Disease: Biological and Behavioral Correlates and Health-Related Quality of Life. *Journal of Paediatric Oncology Nursing: Official Journal of the Association of Pediatric Oncology Nurses, 31*(1), 6. https://doi.org/10.1177/1043454213514632
5. Anderson, L. M., Allen, T. M., Thornburg, C. D., & Bonner, M. J. (2015). Fatigue in Children With Sickle Cell Disease: Association With Neurocognitive and Social-Emotional Functioning and Quality of Life. *Journal of Paediatric Hematology/Oncology, 37*(8), 584. https://doi.org/10.1097/MPH.0000000000000431
6. Badawy, S. M., Barrera, L., Cai, S., & Thompson, A. A. (2018). Association between Participants’ Characteristics, Patient-Reported Outcomes, and Clinical Outcomes in Youth with Sickle Cell Disease. *BioMed Research International, 2018*, e8296139. https://doi.org/10.1155/2018/8296139
7. Badawy, S. M., Thompson, A. A., Holl, J. L., Penedo, F. J., & Liem, R. I. (2018). Healthcare utilization and hydroxyurea adherence in youth with sickle cell disease. *Paediatric Hematology and Oncology, 35*(5‑6), 297‑308. https://doi.org/10.1080/08880018.2018.1505988
8. Badawy, S. M., Thompson, A. A., Lai, J., Penedo, F. J., Rychlik, K., & Liem, R. I. (2017). Health‐related quality of life and adherence to hydroxyurea in adolescents and young adults with sickle cell disease. *Paediatric Blood & Cancer, 64*(6), e26369. https://doi.org/10.1002/pbc.26369
9. Badawy, S. M., Thompson, A. A., Penedo, F. J., Lai, J., Rychlik, K., & Liem, R. I. (2017). Barriers to hydroxyurea adherence and health‐related quality of life in adolescents and young adults with sickle cell disease. *European Journal of Haematology, 98*(6), 608‑614. https://doi.org/10.1111/ejh.12878
10. Bakshi, N., Ross, D., & Krishnamurti, L. (2018). Presence of pain on three or more days of the week is associated with worse patient reported outcomes in adults with sickle cell disease. *Journal of Pain Research, 11*, 313‑318. https://doi.org/10.2147/JPR.S150065
11. Dampier, C., Barry, V., Gross, H. E., Lui, Y., Thornburg, C. D., DeWalt, D. A., & Reeve, B. B. (2016). Initial Evaluation of the Paediatric PROMIS® Health Domains in Children and Adolescents With Sickle Cell Disease. *Paediatric Blood & Cancer, 63*(6), 1031–1037. https://doi.org/10.1002/pbc.25944
12. Dampier, C., Jaeger, B., Gross, H. E., Barry, V., Edwards, L., Lui, Y., DeWalt, D. A., & Reeve, B. B. (2016). Responsiveness of PROMIS ® Paediatric Measures to Hospitalizations for Sickle Pain and Subsequent Recovery. *Paediatric Blood & Cancer, 63*(6), 1038–1045. https://doi.org/10.1002/pbc.25931
13. Dampier, C., Lieff, S., LeBeau, P., Rhee, S., McMurray, M., Rogers, Z., Smith-Whitley, K., & Wang, W. (2010). Health-Related Quality of Life in Children with Sickle Cell Disease: A Report from the Comprehensive Sickle Cell Centers Clinical Trial Consortium. *Paediatric Blood & Cancer, 55*(3), 485. https://doi.org/10.1002/pbc.22497
14. DeWalt, D. A., Gross, H. E., Gipson, D. S., Selewski, D. T., DeWitt, E. M., Dampier, C. D., Hinds, P. S., Huang, I.— C., Thissen, D., & Varni, J. W. (2015). PROMIS® pediatric self-report scales distinguish subgroups of children within and across six common pediatric chronic health conditions. *Quality of Life Research, 24*(9), 2195–2208. https://doi.org/10.1007/s11136-015-0953-3
15. Dougherty, K. A., Schall, J. I., Bertolaso, C., Smith-Whitley, K., & Stallings, V. A. (2020). Vitamin D Supplementation Improves Health-Related Quality of Life and Physical Performance in Children with Sickle Cell Disease and in Healthy Children. *Journal of Paediatric Health Care, 34*(5), 424‑434. https://doi.org/10.1016/j.pedhc.2020.04.007
16. Hildenbrand, A. K., Quinn, C. T., Mara, C. A., Peugh, J. L., McTate, E. A., Britto, M. T., & Crosby, L. E. (2019). A preliminary investigation of the psychometric properties of PROMIS® scales in emerging adults with sickle cell disease. *Health Psychology, 38*(5), 386–390. https://doi.org/10.1037/hea0000696
17. Keller, S., Yang, M., Treadwell, M. J., & Hassell, K. L. (2017). Sensitivity of alternative measures of functioning and wellbeing for adults with sickle cell disease: Comparison of PROMIS® to ASCQ-MeSM. *Health and Quality of Life Outcomes, 15*(1), 117. https://doi.org/10.1186/s12955-017-0661-5
18. Knisely, M. R., Pugh, N., Kroner, B., Masese, R., Gordeuk, V., King, A. A., Smith, S. M., Gurney, J. G., Adams, R., Wun, T., Snyder, A., Glassberg, J., Shah, N., Treadwell, M., & Consortium, S. C. D. I. (2020). Patient-reported outcomes in sickle cell disease and association with clinical and psychosocial factors: Report from the sickle cell disease implementation consortium. *American Journal of Hematology, 95*(9), 1066–1074. https://doi.org/10.1002/ajh.25880
19. Lyon, D., McCain, N., Elswick, R. K., Sturgill, J., Ameringer, S., Jallo, N., Menzies, V., Robins, J., Starkweather, A., Walter, J., & Grap, M. J. (2014). Biobehavioral examination of fatigue across populations: Report from a P30 Center of Excellence. *Nursing Outlook, 62*(5), 322–331. https://doi.org/10.1016/j.outlook.2014.06.008
20. Mason, S. F., Dasgupta, M., Flynn, K. E., Simpson, P. M., & Singh, A. (2023). Comparison of CAT and short forms for PROMIS pain and physical health domains in children with sickle cell disease. *Journal of Patient-Reported Outcomes, 7*, 12. https://doi.org/10.1186/s41687-023-00553-3
21. McGill, L. S., Hughes, A. J., Carroll, C. P., & Bediako, S. M. (2023). Illness Intrusiveness in Adults with Sickle Cell Disease: The Role of Fatigue. *Journal of Clinical Psychology in Medical Settings, 30*(4), 866–875. https://doi.org/10.1007/s10880-023-09950-8
22. Mucalo, L., Field, J. J., Highland, J., Khan, H., Hankins, J. S., Singh, A., & Brandow, A. M. (2023). Preliminary construct validity of patient-reported outcomes to assess chronic pain in adults with sickle cell disease. *Blood Advances, 7*(14), 3658–3665. https://doi.org/10.1182/bloodadvances.2023009707
23. Panepinto, J. A., Paul Scott, J., Badaki-Makun, O., Darbari, D. S., Chumpitazi, C. E., Airewele, G. E., Ellison, A. M., Smith-Whitley, K., Mahajan, P., Sarnaik, S. A., Charles Casper, T., Cook, L. J., Leonard, J., Hulbert, M. L., Powell, E. C., Liem, R. I., Hickey, R., Krishnamurti, L., Hillery, C. A., … for the Paediatric Emergency Care Applied Research Network (PECARN). (2017). Determining the longitudinal validity and meaningful differences in HRQL of the PedsQLTM Sickle Cell Disease Module. *Health and Quality of Life Outcomes, 15*(1), 124. https://doi.org/10.1186/s12955-017-0700-2
24. Panepinto, J. A., Torres, S., Bendo, C. B., McCavit, T. L., Dinu, B., Sherman-Bien, S., Bemrich-Stolz, C., & Varni, J. W. (2014). PedsQLTM multidimensional fatigue scale in sickle cell disease: Feasibility, reliability, and validity. *Paediatric Blood & Cancer, 61*(1), 171–177. https://doi.org/10.1002/pbc.24776
25. Rogers, V., & Lance, E. (2017). Sleep, fatigue and neurodevelopmental outcomes in pediatric sickle cell disease. *Austin Journal of Pediatrics, 4*, 1056–1062.
26. Semko, J. H., Longoria, J., Porter, J., Potter, B., Bhatia, S., Pan, H., Hankins, J. S., & Heitzer, A. M. (2023). Examining the influence of pain and fatigue on neurocognitive functioning in adolescents and young adults with sickle cell disease. *Paediatric Blood & Cancer, 70*(11), e30621. https://doi.org/10.1002/pbc.30621
27. Van Der Land, V., Hijmans, C. T., De Ruiter, M., Mutsaerts, H. J. M. M., Cnossen, M. H., Engelen, M., Majoie, C. B. L. M., Nederveen, A. J., Grootenhuis, M. A., & Fijnvandraat, K. (2015). Volume of white matter hyperintensities is an independent predictor of intelligence quotient and processing speed in children with sickle cell disease. *British Journal of Haematology, 168*(4), 553‑556. https://doi.org/10.1111/bjh.13179
28. Wang, Y., Wang, D. D., Pucka, A. Q., O’Brien, A. R. W., Harte, S. E., & Harris, R. E. (2024). Differential clinical characteristics across traditional Chinese medicine (TCM) Syndromes in patients with sickle cell disease. *Frontiers in Pain Research, 4*, 1233293. https://doi.org/10.3389/fpain.2023.1233293
